# Supplementary material for: Compact meta-differentiator for achieving isotropically high-contrast ultrasonic imaging
Source: Nat Commun. 2024 Apr 5;15:2934. doi: 10.1038/s41467-024-47303-7 (PMC10995138; doi:10.1038/s41467-024-47303-7)
Supplement: Supplementary file 1 — Supplementary Information [file 41467_2024_47303_MOESM1_ESM.pdf]

Supplementary Information for Compact meta-differentiator for achieving isotropically high-contrast ultrasonic imaging

Yurou Jia,[1,2] Suying Zhang,[1] Xuan Zhang,[1] Houyou Long,[1,*] Caibin Xu,[3] Yechao Bai,[4] Ying Cheng,[1,5,†] Dajian Wu,[6] Mingxi Deng,[3] Cheng-Wei Qiu,[2] and Xiaojun Liu[1,5,‡]

[1]*Department of Physics, Collaborative Innovation Center of Advanced Microstructures, Nanjing University, Nanjing 210093, China*
[2]*Department of Electrical and Computer Engineering, National University of Singapore, Singapore 117583, Singapore.*
[3]*College of Aerospace Engineering, Chongqing University, Chongqing 400044, China*
[4]*School of Electronic Science and Engineering, Nanjing University, Nanjing, 210023, China*
[5]*State Key Laboratory of Acoustics, Institute of Acoustics, Chinese Academy of Sciences, Beijing 100190, China*
[6]*Jiangsu Key Lab on Opto-Electronic Technology, School of Physics and Technology, Nanjing Normal University, Nanjing 210023, China*

(Dated: March 14, 2024)

* Corresponding author: longhouyou@nju.edu.cn
† Corresponding author: chengying@nju.edu.cn
‡ Corresponding author: liuxiaojun@nju.edu.cn

CONTENTS

Supplementary Note 1. Direct Fourier transform based on focus phase factor 3
Supplementary Note 2. Output image obtained from convolution operation 4
Supplementary Note 3. Spatial wavevector redistribution 5
Supplementary Note 4. Theoretical analysis based on angular spectrum method 6
Supplementary Note 5. Edge enhancement of objects utilizing convolution method 7
Supplementary Note 6. Acoustic metasurface carrying other topological charges 8
Supplementary Note 7. 1D edge detection and vertex detection 9
Supplementary Note 8. Isotropic edge detection of complex amplitude objects 10
Supplementary Note 9. Imaging resolution of 2D spatial differentiator 11
Supplementary Note 10. Frequency response of acoustic meta-differentiator 13
Supplementary Note 11. Edge detection of objects placed at different positions 14
Supplementary Note 12. Effects of amplitude and phase discretization on 2D spatial differentiation 15
Supplementary Note 13. Cross-sectional view of acoustic meta-differentiator 16
Supplementary Note 14. Experimental results for amplitude object in different size 17
Supplementary Note 15. Experimental results for amplitude object in different shape 18
Supplementary Note 16. Demonstration of Reflective-mode Meta-differentiator 19
Supplementary Note 17. Reflective imaging system utilizing transmission-mode meta-differentiator 20
Supplementary Note 18. Imaging of phase objects with different phase gradients 21
Supplementary Note 19. Control experiments on object imaging with and without acoustic meta-differentiator 22
Supplementary Note 20. Isotropic edge-enhanced imaging of complex phase objects 23
Supplementary Note 21. Experimental results for phase object in different shape 24
Supplementary Note 22. Comparison between focus metasurface and 2D spatial differentiator 25
Supplementary Note 23. Edge-enhanced imaging of 3D mini hands 26
Supplementary Note 24. 3D object imaging based on acoustic meta-differentiator 27
Supplementary Note 25. Comparison among prior acoustic meta-differentiators 28

References 30

Supplementary Note 1. Direct Fourier transform based on focus phase factor

To verify the functionality of the focus phase factor, we consider a point source with an acoustic field $p_1(x_1, y_1)$ located at the input plane, and impinging through the compact acoustic metasurface with an aperture function $t_{\text{meta}}(x', y')$. The acoustic field $p_2(x_2, y_2)$ at the output plane is calculated using the Rayleigh-Sommerfeld diffraction integral:

$$p_2\left(x_2, y_2\right) = -\frac{k^2}{4\pi^2} \iint p_1\left(x_1, y_1\right) \frac{e^{ikr_1}}{r_1} t_{\text{meta}}\left(x', y'\right) \frac{e^{ikr_2}}{r_2} \mathrm{d}x' \mathrm{d}y', \tag{1}$$

where k represents the wave vector, and $r_{1,2} = \sqrt{(x' - x_{1,2})^2 + (y' - y_{1,2})^2 + f^2}$ denote the distances between two adjacent planes with a spacing interval of f. Under the paraxial approximation in the Fresnel regime, the distance terms r_1 and r_2 can be simplified and translated into:

$$r_1 = f\left[1 + \frac{\left(x' - x_1\right)^2 + \left(y' - y_1\right)^2}{2f^2}\right], r_2 = f\left[1 + \frac{\left(x' - x_2\right)^2 + \left(y' - y_2\right)^2}{2f^2}\right]. \tag{2}$$

Next, we substitute Eq. (2) into Eq. (1), and the acoustic field $p_2(x_2, y_2)$ at the output plane can be rewritten as:

$$\begin{aligned} p_2\left(x_2, y_2\right) &= -\frac{k^2}{4\pi^2} \iint p_1\left(x_1, y_1\right) \frac{e^{ikf\left[1 + \frac{\left(x' - x_1\right)^2 + \left(y' - y_1\right)^2}{2f^2}\right]}}{f} t_{\text{meta}}\left(x', y'\right) \frac{e^{ikf\left[1 + \frac{\left(x' - x_2\right)^2 + \left(y' - y_2\right)^2}{2f^2}\right]}}{f} \mathrm{d}x' \mathrm{d}y' \\ &= -\frac{k^2}{4\pi^2} \frac{e^{i2kf}}{f^2} \iint p_1\left(x_1, y_1\right) e^{\frac{ik}{2f}\left[\left(x' - x_1\right)^2 + \left(y' - y_1\right)^2 + \left(x_2 - x'\right)^2 + \left(y_2 - y'\right)^2\right]} t_{\text{meta}}\left(x', y'\right) \mathrm{d}x' \mathrm{d}y' \\ &= -\frac{k^2}{4\pi^2} \frac{e^{i2kf}}{f^2} e^{\frac{ik}{2f}\left(x_2^2 + y_2^2 + x_1^2 + y_1^2\right)} p_1\left(x_1, y_1\right) \iint e^{\frac{-ik}{f}\left[\left(x_1 + x_2\right)x' + \left(y_1 + y_2\right)y'\right]} e^{\frac{ik}{f}\left(x'^2 + y'^2\right)} t_{\text{meta}}\left(x', y'\right) \mathrm{d}x' \mathrm{d}y'. \end{aligned} \tag{3}$$

Considering the Fourier transform function in the form of $e^{-i(kx' + ky')}$, the redundant term $e^{\frac{ik}{f}\left(x'^2 + y'^2\right)}$ in Eq. (3) should be eliminated. In this way, we introduce the focus phase factor $e^{-i2k\left(\sqrt{x'^2 + y'^2 + f^2} - f\right)}$ into the aperture function $t_{\text{meta}}(x', y')$ as:

$$t_{\text{meta}}(x', y') = e^{-i2k\left(\sqrt{x'^2 + y'^2 + f^2} - f\right)} t_{\text{tran}}(x', y'). \tag{4}$$

Here, $t_{\text{tran}}(x', y')$ denotes the transfer function associated with mathematical operations in the spatial Fourier domain. Under the paraxial approximation, the focus phase is transformed into a hyperbolic phase:

$$t_{\text{meta}}\left(x', y'\right) = e^{-\frac{ik}{f}\left(x'^2 + y'^2\right)} t_{\text{tran}}(x', y'). \tag{5}$$

As a result, the reformulated output acoustic field $p_2(x_2, y_2)$ becomes:

$$\begin{aligned} p_2\left(x_2, y_2\right) &= -\frac{k^2}{4\pi^2} \frac{e^{i2kf}}{f^2} e^{\frac{ik}{2f}\left(x_2^2 + y_2^2 + x_1^2 + y_1^2\right)} p_1\left(x_1, y_1\right) \iint t_{\text{tran}}(x', y') e^{\frac{-ik}{f}\left[\left(x_1 + x_2\right)x' + \left(y_1 + y_2\right)y'\right]} \mathrm{d}x' \mathrm{d}y' \\ &= -\frac{k^2}{4\pi^2} \frac{e^{i2kf}}{f^2} e^{\frac{ik}{2f}\left(x_2^2 + y_2^2 + x_1^2 + y_1^2\right)} p_1\left(x_1, y_1\right) \mathscr{F}\left\{t_{\text{tran}}(x', y')\right\}_{\left[k_x = \frac{k(x_1 + x_2)}{f}, k_y = \frac{k(y_1 + y_2)}{f}\right]}, \end{aligned} \tag{6}$$

in which $\mathscr{F}$ represents the Fourier transform operation, and $k_x = \frac{k(x_1 + x_2)}{f}$ and $k_y = \frac{k(y_1 + y_2)}{f}$ are the spatial wavevectors along the x- and y-axes, respectively. From Eq. (6), it can be observed that the acoustic metasurface, imparted with $t_{\text{meta}}(x', y')$, directly modulates the spatial Fourier spectrum for the desired outputs by superimposing the focus phase factor onto the transfer function [1, 2].

Supplementary Note 2. Output image obtained from convolution operation

Instead of a point source, we consider an input object with an acoustic field $p_1(x_1, y_1)$ transmitted through the acoustic metasurface with an aperture function $t_{\mathrm{meta}}(x', y')$. The resulting image with the acoustic field $p_2(x_2, y_2)$ at the output plane can be expressed as:

$$p_2(x_2, y_2) = -\frac{k^2}{4\pi^2} \iint \left[\iint p_1(x_1, y_1) \frac{e^{ikr_1}}{r_1} \mathrm{d}x_1 \mathrm{d}y_1 \right] t_{\mathrm{meta}}(x', y') \frac{e^{ikr_2}}{r_2} \mathrm{d}x' \mathrm{d}y'. \tag{7}$$

Under the paraxial approximation, Eq. (7) is transformed into:

$$\begin{aligned} p_2(x_2, y_2) &= -\frac{k^2}{4\pi^2} \frac{1}{f^2} \iint \left\{ \iint p_1(x_1, y_1) e^{ikf\left[1+\frac{(x'-x_1)^2+(y'-y_1)^2}{2f^2}\right]} \mathrm{d}x_1 \mathrm{d}y_1 \right\} t_{\mathrm{meta}}(x', y') e^{ikf\left[1+\frac{(x_2-x')^2+(y_2-y')^2}{2f^2}\right]} \mathrm{d}x' \mathrm{d}y' \\ &= -\frac{k^2}{4\pi^2} \frac{e^{ik\left(2f+\frac{x_2^2+y_2^2}{2f}\right)}}{f^2} \iint \left\{ \iint p_1(x_1, y_1) e^{ik\frac{x_1^2+y_1^2}{2f}} \mathrm{d}x_1 \mathrm{d}y_1 \right\} t_{\mathrm{meta}}(x', y') e^{ik\frac{x'^2+y'^2}{f}} e^{-ik\frac{(x_1+x_2)x'+(y_1+y_2)y'}{f}} \mathrm{d}x' \mathrm{d}y'. \end{aligned} \tag{8}$$

By introducing the aperture function $t_{\mathrm{meta}}(x', y') = e^{-\frac{ik}{f}(x'^2+y'^2)} t_{\mathrm{tran}}(x', y')$ into Eq. (8), the output image $p_2(x_2, y_2)$ can be calculated as:

$$\begin{aligned} p_2(x_2, y_2) &= -\frac{k^2}{4\pi^2} \frac{e^{ik\left(2f+\frac{x_2^2+y_2^2}{2f}\right)}}{f^2} \iint \left\{ \iint p_1(x_1, y_1) e^{ik\frac{x_1^2+y_1^2}{2f}} \mathrm{d}x_1 \mathrm{d}y_1 \right\} t_{\mathrm{tran}}(x', y') e^{-ik\frac{(x_1+x_2)x'+(y_1+y_2)y'}{f}} \mathrm{d}x' \mathrm{d}y' \\ &= -\frac{k^2}{4\pi^2} \frac{e^{ik\left(2f+\frac{x_2^2+y_2^2}{2f}\right)}}{f^2} \iint p_1(x_1, y_1) e^{ik\frac{x_1^2+y_1^2}{2f}} \mathscr{F}\left\{ t_{\mathrm{tran}}(x', y') \right\}_{\left[k\frac{(x_1+x_2)}{f}, k\frac{(y_1+y_2)}{f}\right]} \mathrm{d}x_1 \mathrm{d}y_1, \end{aligned} \tag{9}$$

where the symbol $\mathscr{F}$ correspond to the Fourier transform operation, and $k_x = \frac{k(x_1+x_2)}{f}$, $k_y = \frac{k(y_1+y_2)}{f}$ represent the spatial wavevectors along the x- and y-axes, respectively. According to the convolution theorem, Eq. (9) can be reformulated as:

$$p_2(x_2, y_2) = -\frac{k^2}{4\pi^2} \frac{e^{ik\left(2f+\frac{x_2^2+y_2^2}{2f}\right)}}{f^2} \left\{ p_1(-x_2, -y_2) e^{ik\frac{x_2^2+y_2^2}{2f}} \right\} \otimes \mathscr{F}\left\{ t_{\mathrm{tran}}(x', y') \right\}_{\left[k\frac{x_2}{f}, k\frac{y_2}{f}\right]}, \tag{10}$$

in which the symbol $\otimes$ denotes the 2D convolution operation, and $\mathscr{F}\{t_{\mathrm{tran}}(x', y')\}_{(k_x, k_y)}$ represents the Fourier transform of $t_{\mathrm{tran}}(x', y')$ at the spatial wavevectors of $k_x = \frac{k}{f} x_2$ and $k_y = \frac{k}{f} y_2$ along the x- and y-axes, respectively. Under the paraxial approximation condition, the phase factor $e^{ik\frac{x_2^2+y_2^2}{2f}}$ can be neglected. Therefore, the output image can be obtained by performing a convolution operation between the input object, rotated by 180° about the origin, and the transfer function $t_{\mathrm{tran}}(x', y')$ in the spatial Fourier domain [1, 2].

Supplementary Note 3. Spatial wavevector redistribution

In Supplementary Fig. **1**a, the phase profile imparted onto the acoustic metasurface is a superposition of the focus phase and the spiral phase carrying a 1st-order topological charge (TC). We can then utilize the spatial wavevector redistribution mechanism to explain the 2D spatial differentiation operation achieved by the acoustic metasurface. As depicted in Supplementary Fig. **1**b, the combined effect of the focus and spiral phases results in the generation of wavevectors k_r and k_t along the radial and tangential directions, respectively. The expressions for these wavevectors are given by:

$$\begin{cases} k_r = \left|\dfrac{\mathrm{d}\varphi}{\mathrm{d}r}\right| = k\dfrac{2r}{\sqrt{r^2+f^2}}, \\ k_t = \left|\dfrac{\mathrm{d}\varphi}{\mathrm{d}l}\right| = \left|\dfrac{\mathrm{d}\varphi}{\mathrm{d}\theta}\dfrac{\mathrm{d}\theta}{\mathrm{d}l}\right| = k\dfrac{\lambda}{2\pi r}. \end{cases} \tag{11}$$

Here, dl represents an infinitesimal length along the tangential direction, and λ denotes the acoustic wavelength. In this context, the resultant wavevector $k' = \sqrt{k_r^2 + k_t^2}$ is redirected to a higher value, leaving an isolated central zero in the wavevector space. This phenomenon contributes to the achievement of 2D spatial differentiation operation [3, 4].

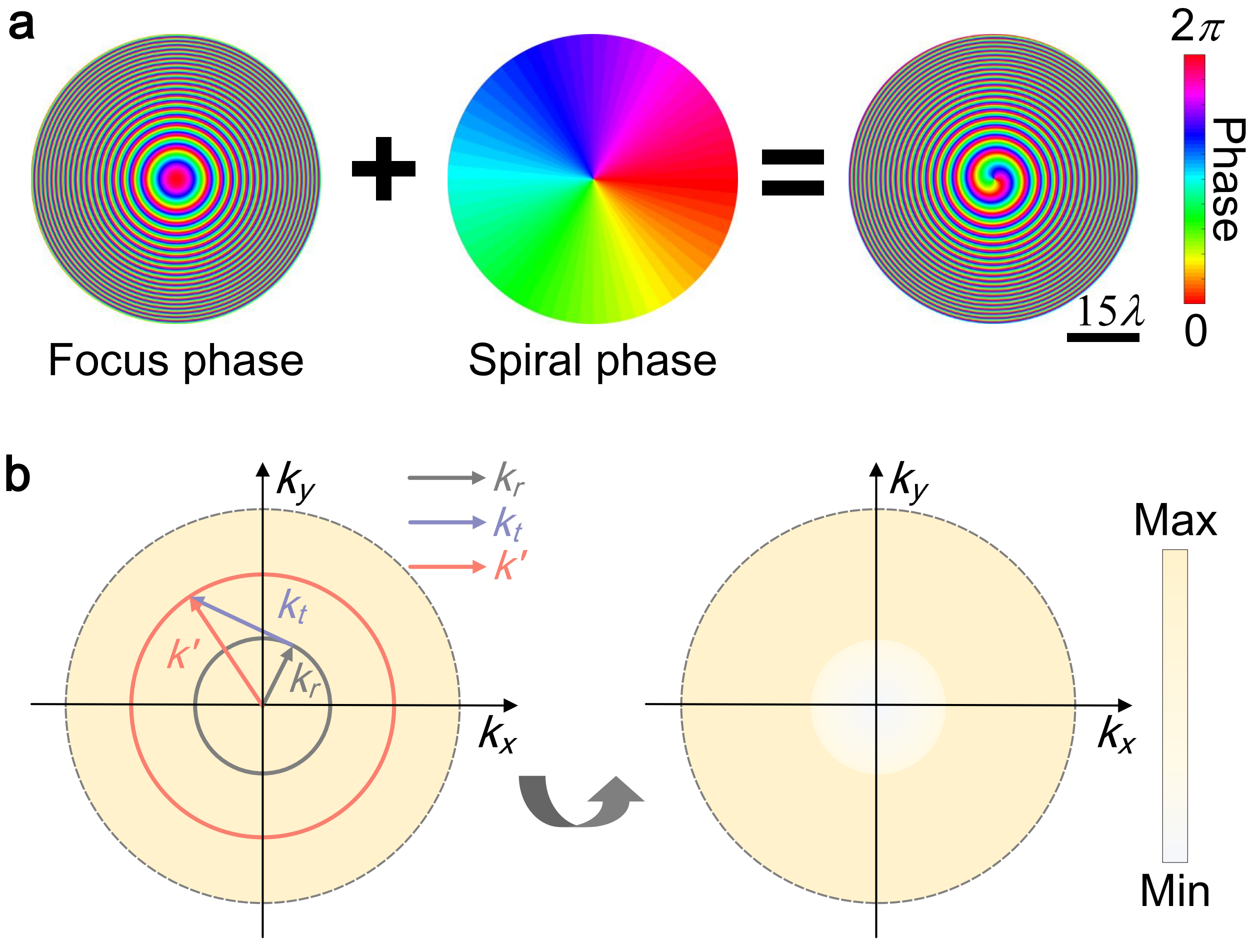

Supplementary Figure 1. **Spatial wavevector redistribution mechanism. a** Phase profile imparted onto the acoustic metasurface, which is a superposition of the focus phase and a 1st-order spiral phase. **b** Schematic illustration of the spatial wavevector redistribution mechanism for achieving 2D spatial differentiation operation.

Supplementary Note 4. Theoretical analysis based on angular spectrum method

The angular spectrum method is a precise technique for modeling the propagation of acoustic waves by decomposing wave fields within a single plane into multiple plane wave components through the 2D spatial Fourier transform [5]. Each plane wave component is then propagated to a destination plane in the Fourier domain, and the wave fields are reconstructed using the inverse spatial Fourier transform. To perform these forward and inverse Fourier transforms efficiently, the fast Fourier transform algorithm is employed, offering a fast, simple, and efficient computational framework. As shown in Supplementary Fig. **2**, at the plane $z = z_1$, the angular spectrum of the acoustic field $p_1(x, y, z_1)$ is obtained from the Fourier transform as:

$$P_1(k_x, k_y, z_1) = \iint p_1(x, y, z_1)e^{-i(k_x x + k_y y)}\mathrm{d}x\mathrm{d}y \tag{12}$$

Once $P_1(x, y, z_1)$ is known, the angular spectrum of the acoustic field at any arbitrary plane z can be computed by multiplying it with the propagator function as:

$$P(k_x, k_y, z) = P_1(k_x, k_y, z_1)H(k_x, k_y, z - z_1), \tag{13}$$

where $H(k_x, k_y, z) = e^{ik_z(z-z_1)}$ is the propagator function with $k_z = \sqrt{k^2 - k_x^2 - k_y^2}$. To obtain the real-space acoustic field $p_2(x, y, z_2)$ at the plane $z = z_2$, we perform the inverse Fourier transform of $P_2(k_x, k_y, z_2)$, resulting in:

$$p_2(x, y, z_2) = \frac{1}{4\pi^2}\iint P_1(k_x, k_y, z_1)H(k_x, k_y, z_2 - z_1)\mathrm{d}k_x\mathrm{d}k_y \tag{14}$$

In this study, we consider the case where $z_1 = -f$ and $z_2 = 0$, representing the scenario where the input object with the acoustic field p_1 propagates to the front surface of the acoustic metasurface, leading to the generation of the acoustic field p_2. Given the relatively small thickness of the acoustic metasurface, we obtain the acoustic field p_3 at the back surface of acoustic metasurface by multiplying p_2 with the aperture function $t_{\mathrm{meta}}(x', y')$. Finally, based on the angular spectrum method, the acoustic field p_4 at the output plane can be accessed.

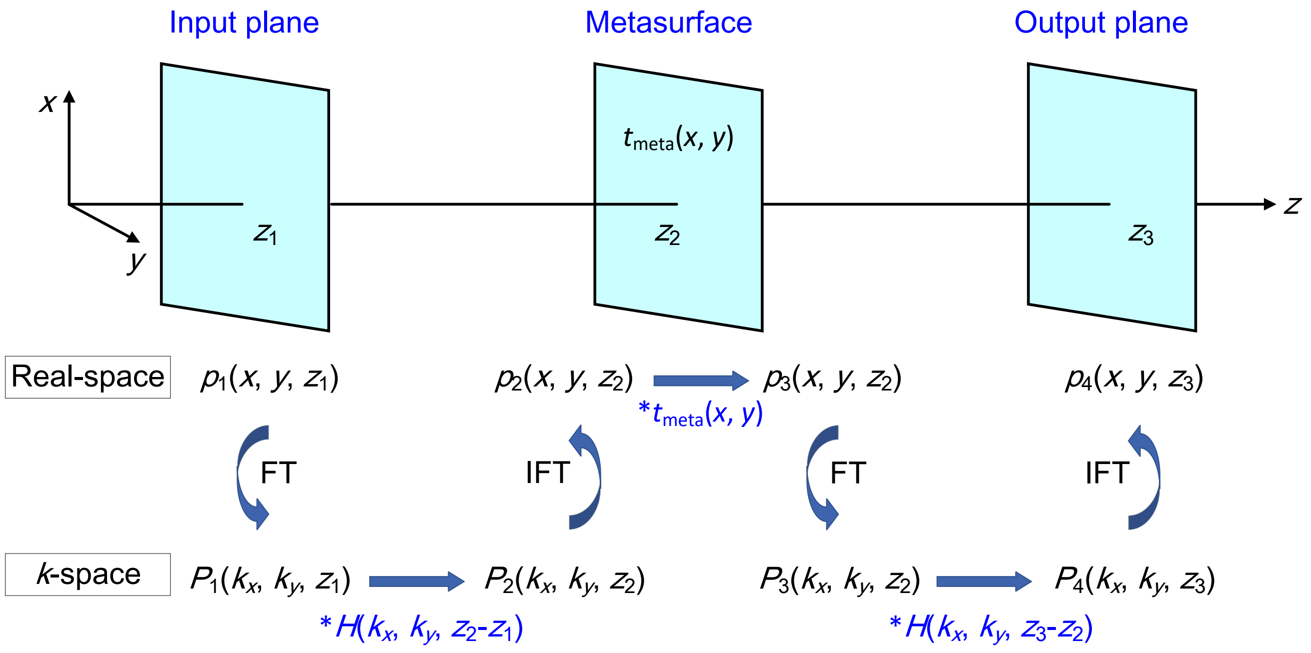

Supplementary Figure 2. **Acoustic field reconstructions based on angular spectrum method.** The symbols FT and IFT denote the Fourier transform and inverse Fourier transform operations, respectively.

Supplementary Note 5. Edge enhancement of objects utilizing convolution method

In Supplementary Fig. **3**a, we present the acoustic intensity and phase profiles of an ideal PSF for the imaging system, illustrating a doughnut-shaped intensity ring with a phase distribution ranging from 0 to 2π around the ring within a $2\lambda\times2\lambda$ region. Supplementary Figure **3**b introduces an amplitude object in the form of a 'Panda' with dimensions of $60\lambda\times60\lambda$, where the white region represents full transmission of acoustic waves, while the black area indicates no transmission. By leveraging the convolution process of the PSF with the amplitude object, we present the resulting acoustic intensity and phase profiles of the output image in Supplementary Fig. **3**c. In 'flat' regions, neighboring points exhibit doughnut rings with identical phase and amplitude characteristics, leading to destructive interference due to the π phase difference across the doughnut [6]. Conversely, at the edges of the object, the PSF of neighboring points deviates either in intensity or phase retardation, resulting in a brightening effect along the edges. Therefore, both the intensity and phase profiles collaboratively enhance all edges of the 'Panda' against the background for clearer recognition.

We further delve into the analysis of another amplitude object in the form of a 'Tree', as depicted in Supplementary Fig. **3**d. The corresponding intensity and phase profiles of the output image, processed via the convolution method, are presented in Supplementary Fig. **3**e. In this scenario, the phase profile effectively conveys edge information of objects, similar to the role played by the intensity profile. Furthermore, the non-uniformity in phase along the edges generates an apparent shadow effect [7]. Additionally, we discuss the convoluted results based on the PSF within the area of $18\lambda\times18\lambda$ with these complex objects, as shown in Supplementary Figs. **3**f-j. From the output images, the normalized intensity profiles clearly reveal the full edges of objects in a more distinct manner. Although the phase profile is more complex due to the multiple-ring spiral phase in the PSF, the edges can still be discerned along the phase profile with a wider width. Consequently, the edge information of objects can be more easily extracted from the intensity profile than the phase profile. To enhance the utility of phase profile for object recognition, optimizing the PSF of imaging system by eliminating the outer-ring phase profiles is necessary.

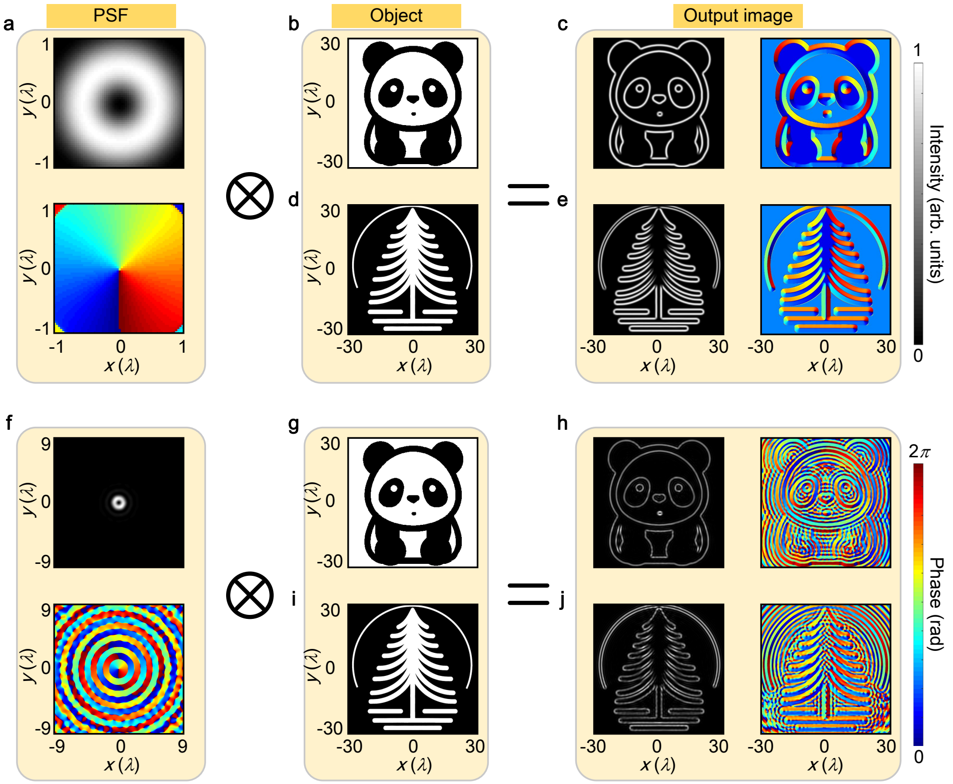

Supplementary Figure 3. **Convolution of amplitude objects with the point spread function (PSF) of imaging system. a** Acoustic intensity and phase distributions of the PSF within $2\lambda\times2\lambda$ region. **b** Amplitude object in the shape of a 'Panda'. **c** Acoustic intensity and phase distributions of the output image calculated from the convolution of the PSF with the amplitude object. **d-e** Equivalent to (**b**)-(**c**), but for the amplitude object in the form of a 'Tree'. The images of 'Panda' and 'Tree' were hand-painted by Yurou Jia, who is the first author of this work. **f-j** Same as (**a**)-(**e**), but for the results based on the PSF within a region of $18\lambda\times18\lambda$.

Supplementary Note 6. Acoustic metasurface carrying other topological charges

The acoustic metasurface, composed of a focus phase and a spiral phase carrying a 1st-order TC, has been demonstrated to exhibit high-performance edge enhancement behavior in the manuscript. When the transfer function is set as $t_{\mathrm{tran}}(r,\theta) = re^{-i\theta}$ with a -1st-order TC, the amplitude and phase profiles imprinted onto the acoustic metasurface are given by $A = |r|$ and $\varphi(r,\theta) = -2k\left(\sqrt{r^2+f^2}-f\right) - \theta$, respectively. Here, the focal length f is set to be 40λ at an operating frequency of 1 MHz, where λ is 1.5 mm. As depicted in Supplementary Fig. **4**a, the amplitude profile exhibits linear transmission in the radial direction within a radius of 30λ, while the spiral phase profile rotates clockwise with a rotation number of 20. To illustrate the edge enhancement capability of this acoustic metasurface, an amplitude object in the shape of the numeral 2 is placed at the input plane $z = -f$. The object has a height of 6λ, a width of 3.75λ, and a slit of 0.75λ. The calculated acoustic intensity and phase distributions at the output plane $z = f$ are presented in Supplementary Fig. **4**b, using the angular spectrum method. It can be observed that all edges of the numeral 2 are enhanced with isotropic intensity, and its phase profile exhibits a π difference between two symmetric positions about the origin. Supplementary Figure **4**c shows the intensity profiles along the vertical axis of the input (solid line) and output (dashed line) images, revealing the edge enhancements achieved by the acoustic metasurface with a -1st-order TC.

To investigate the influence of higher-order TCs, we consider an acoustic metasurface carrying a TC of 2, with an amplitude profile given by $A = r^2$ and a phase profile $\varphi(\theta) = -2k\left(\sqrt{r^2+f^2}-f\right) + 2\theta$, as shown in Supplementary Fig. **4**d. The corresponding acoustic intensity and phase distributions at the output plane are presented in Supplementary Fig. **4**e. Interestingly, the intensity-enhanced regions are not precisely located at the edges of the input objects, as indicated by the intensity profiles along the vertical direction of input and output images in Supplementary Fig. **4**f. Consequently, only acoustic metasurfaces with TCs of ± 1 are effective for isotropic edge detection [6].

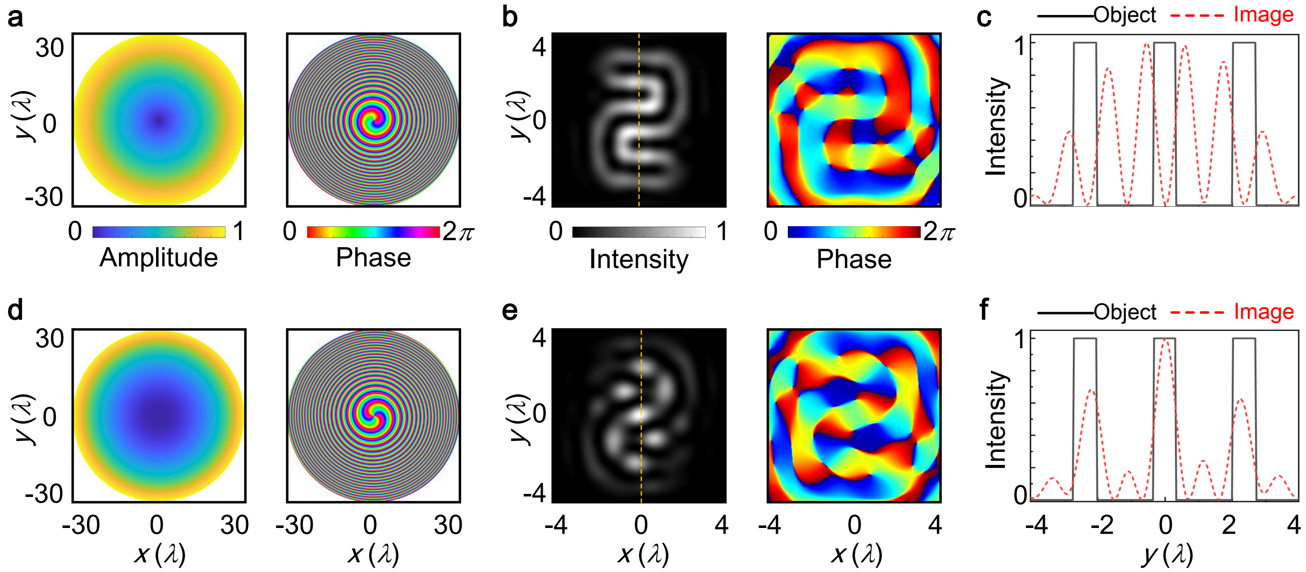

Supplementary Figure 4. **Performance of acoustic metasurfaces carrying other TCs.** **a** Amplitude and phase profiles imprinted onto the acoustic metasurface with a TC of -1. **b** Calculated acoustic intensity and phase distributions at the output plane. **c** Intensity profiles along the vertical direction of both the input and output images. **d**-**f** Same as (**a**)-(**c**) but for the acoustic metasurface carrying a TC of 2.

Supplementary Note 7. 1D edge detection and vertex detection

Edge detection, which is based on significant changes in signal derivatives due to inhomogeneous mediums, is a widely used method for characterizing object boundaries. The common approach for edge detection involves utilizing the -1st-order derivative operation ($\left|\frac{\mathrm{d}p_1(x_1)}{\mathrm{d}x_1}\right|$ or $\left|\frac{\mathrm{d}p_1(y_1)}{\mathrm{d}y_1}\right|$), represented as $t_{\mathrm{tran}}(x') \propto ix'$ or $t_{\mathrm{tran}}(y') \propto iy'$ with respect to the x-axis or y-axis in the spatial Fourier domain [1]. Supplementary Figure **5**a illustrates the amplitude and phase profiles derived from the transfer function $t_{\mathrm{tran}}(x') \propto ix'$, combined with the focus phase. The amplitude profile exhibits symmetry about the central vertical axis and gradually increases away from the center. The phase distribution shows a π difference between the left and right halves. To demonstrate 1D edge detection along the x-axis, we place a point source at the input plane $z = -f$, transmitting through the acoustic metasurface located at $z = 0$. The calculated acoustic intensity and phase profiles at the output plane $z = f$ are presented in Supplementary Fig. **5**b. The intensity pattern is divided into two halves with a pair of high-intensity spots, while the phase profile displays a binary distribution with π shifts. Next, we consider an amplitude object in the shape of numeral 2 placed at the input plane, with a height of 6λ, a width of 3.75λ, and a slit of 0.75λ. The resulting acoustic intensity and phase distributions at the output plane are shown in Supplementary Fig. **5**c. It can be observed that only the vertical boundaries of the numeral 2 are enhanced, thus confirming the 1D spatial differentiation along the x-axis. Similarly, 1D edge detection along the y-axis can be achieved by replacing the variable x with y. Supplementary Figures **5**d-f demonstrate the acoustic metasurface imprinted with the transfer function $t_{\mathrm{tran}}(y') \propto iy'$, which enables 1D spatial differentiation along the y-axis.

Moreover, we showcase 2D anisotropic vertex detection by multiplying the 1D edge detections along the x-axis and y-axis ($\left|\frac{\mathrm{d}p_1(x_1)}{\mathrm{d}x_1} \times \frac{\mathrm{d}p_1(y_1)}{\mathrm{d}y_1}\right|$) [1]. The amplitude and phase profiles imparted onto the acoustic metasurface are depicted in Supplementary Fig. **5**g. The binary phase distribution is split into four sections with π shifts between adjacent neighbors, while the amplitude profile is divided into four parts along the diagonal directions [8]. Supplementary Figure **5**h displays the calculated intensity and phase profiles of a point source at the output plane. The intensity profile exhibits four high-intensity spots along the clinodiagonal directions, and the phase profile shows a quaternary distribution with π shifts. When considering the input object of the numeral 2, the overlapping parts of the x- and y-directional edges are strengthened at the output plane, as shown in Supplementary Fig. **5**i, thus confirming the functionality of vertex detection. Our 2D spatial differentiator, with its ability to provide isotropic edge-enhanced imaging, excels in presenting the complete boundary information of input objects.

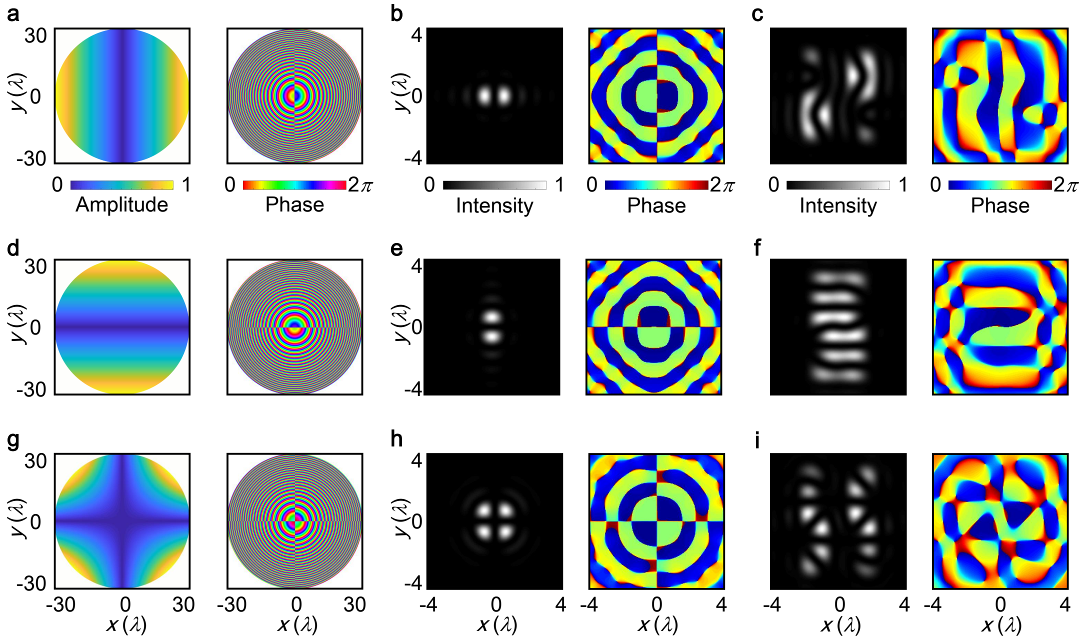

Supplementary Figure 5. **1D edge detection and vertex detection. a** Amplitude and phase profiles derived from the transfer function $t_{\mathrm{tran}}(x') \propto ix'$, combined with the focus phase. Calculated acoustic intensity and phase profiles at the output plane for (**b**) a point source and (**c**) an amplitude object featuring the numeral 2 placed at the input plane. **d-f**, **g-i** Same as (**a**)-(**c**), but using the transfer functions $t_{\mathrm{tran}}(y') \propto iy'$ and $t_{\mathrm{tran}}(y') \propto -x'y'$, respectively.

Supplementary Note 8. Isotropic edge detection of complex amplitude objects

We also investigate the application of 2D spatial differentiators for the isotropic edge detection of complex amplitude objects. The upper panel of Supplementary Figs. **6**a-d displays various amplitude objects representing the numerals 0, 1, 3, and 4. In these objects, white regions indicate full transmission of acoustic waves, while black areas correspond to complete blockage of acoustic waves. The lower panel shows the resulting calculated distributions of acoustic intensity at the output plane. To further illustrate the effectiveness and versatility of the spatial differentiator in isotropic edge enhancements, Supplementary Figs. **6**e-h present input objects depicting the alphabets N, J, U, and S, along with the corresponding output images after processing them with the 2D spatial differentiator. Notably, all boundaries within the different amplitude objects are accentuated with uniform intensity distributions.

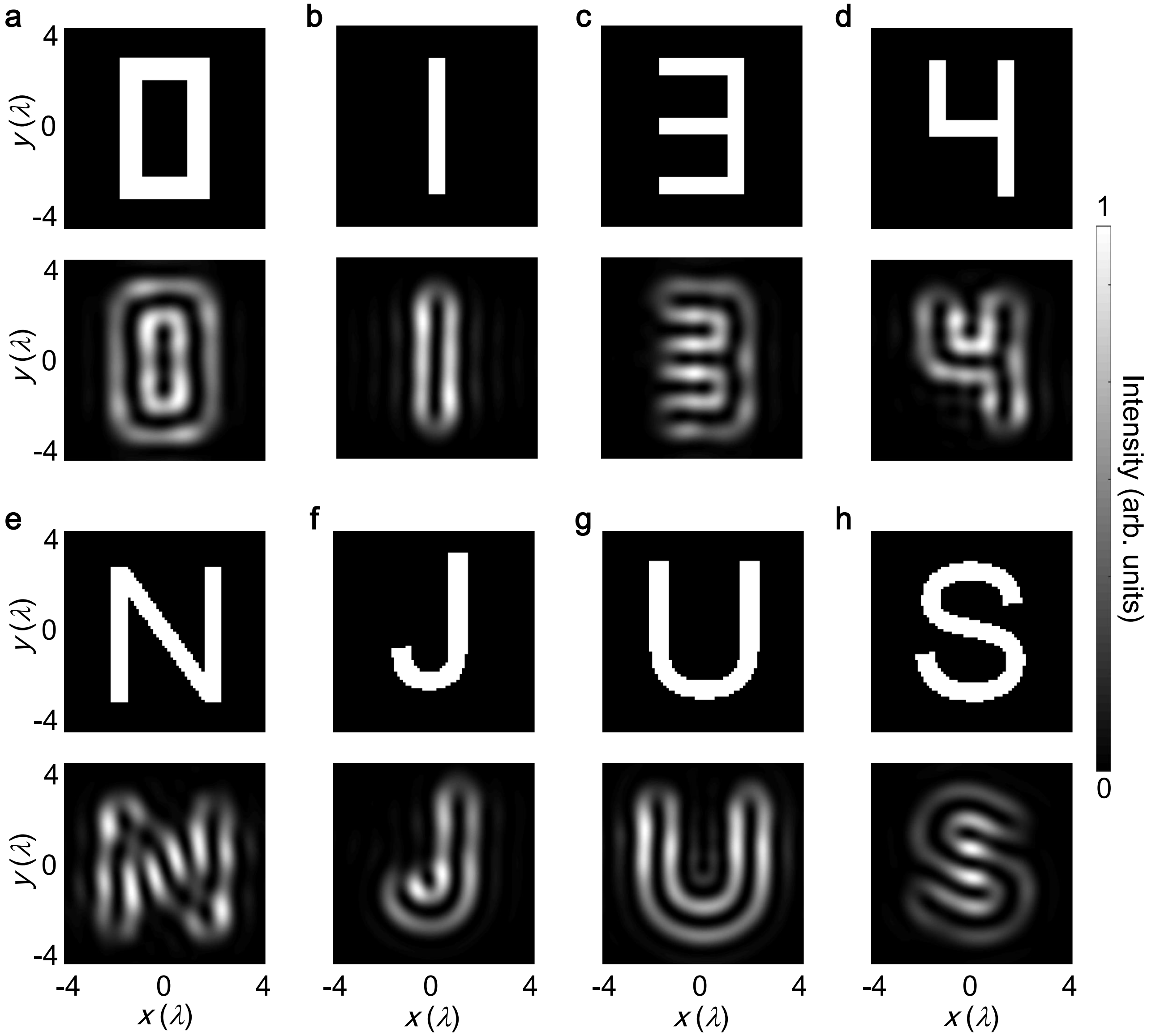

Supplementary Figure 6. **Isotropic edge detection of complex amplitude objects. a-d** Upper panel: amplitude objects with the shapes of the numerals 0, 1, 3, and 4 placed at the input plane. Lower panel: calculated acoustic intensity distributions at the output plane. **e-h** Same as (**a**)-(**d**), but with the amplitude objects depicting the alphabets N, J, U, and S.

Supplementary Note 9. Imaging resolution of 2D spatial differentiator

To assess the imaging resolution of the 2D spatial differentiator, we employ the standard United States Air Force 1951 (USAF 1951) resolution test chart. Supplementary Figure **7**a depicts the amplitude object of the high-resolution USAF 1951 test chart. In this representation, white regions correspond to areas with an acoustic field amplitude of 1 Pa, while black areas indicate the absence of acoustic fields. The calculated intensity profile of the USAF 1951 test chart at the output plane, obtained using the convolution method, is displayed in Supplementary Fig. **7**b. The spatial differentiator operates at a working frequency of 1 MHz in water, resulting in an acoustic wavelength of 1.5 mm. Enlarged views of segments **i**, **ii**, **iii**, and **iv** are provided in Supplementary Figs. **7**c-f, corresponding to the resolutions of 2.475 mm (1.65 λ), 1.725 mm (1.15 λ), 1.2375 mm (0.825 λ), and 1.05 mm (0.7 λ), respectively. Additionally, the lower panel displays intensity profiles along the horizontal direction for segments **i**, **ii**, **iii**, and **iv**. Notably, clear edge enhancements are observed for the three rectangular slots with resolutions ranging from 2.475 mm to 1.2375 mm. However, the imaging quality of the three rectangular slots with a resolution of 1.05 mm is generally poor, characterized by blurred and indistinct edges. Thus, the 2D spatial differentiator demonstrates effective isotropic edge enhancement capabilities, with a maximum achievable resolution of 1.2375 mm at the wavelength of 1.5 mm in the test chart.

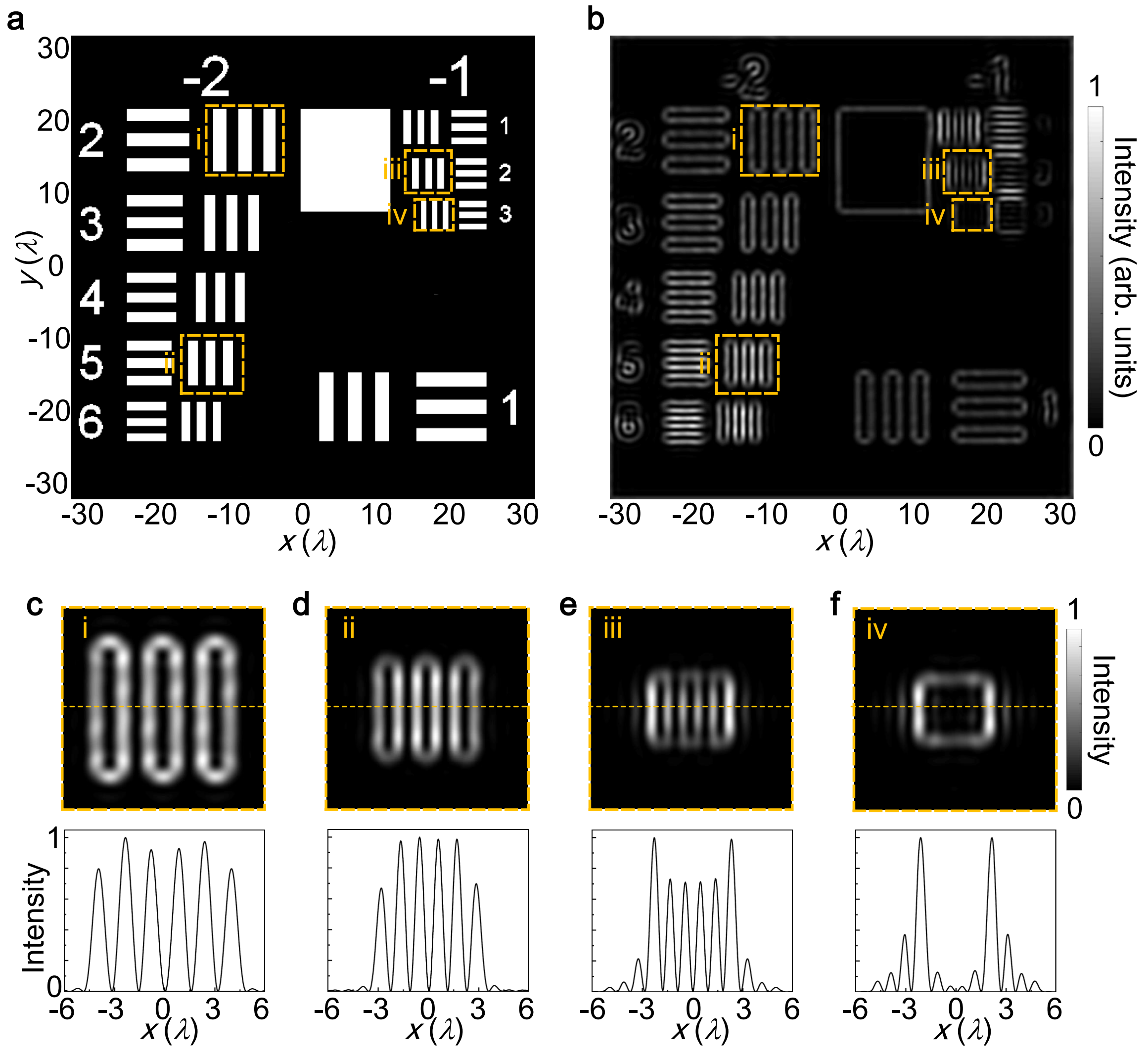

Supplementary Figure 7. **Imaging resolution of 2D spatial differentiator. a** Amplitude object of the United States Air Force 1951 (USAF 1951) resolution test chart. **b** Calculated intensity profile of the USAF1951 test chart at the output plane using the convolution method. **c-f** Upper panel: enlarged views of segments **i**, **ii**, **iii**, and **iv**. Lower panel: intensity lines along the horizontal direction of segments **i**, **ii**, **iii**, and **iv**.

Furthermore, we have addressed the critical resolution of edge detection achieved by the proposed meta-differentiator. In Supplementary Fig. **8**a, the object images are depicted, comprising two rectangular objects with a length of 5λ, a width of 0.75λ, and separation d_s of λ, 0.8λ, 0.75λ, and 0.7λ. In Supplementary Fig. **8**b, the upper panel illustrates the corresponding acoustic intensity distributions at the imaging plane, while the lower panel presents the line-scanning intensity along the horizontal axis indicated by yellow dashed lines. The intensity profile is normalized with respect to the maximum value. Notably, when the separation d_s is λ, all edges of the two rectangles are clearly discernible from the intensity profile. However, as we reduce the separation d_s to 0.8λ or 0.75λ, the edges of both rectangles remain observable, albeit with a slight increase in sidelobe intensity. Upon further reducing the separation d_s to 0.7λ, the intensified sidelobe intensity significantly impacts the edge extraction of inner edges between the two rectangles, rendering it challenging to distinguish between the individual objects. Therefore, the critical resolution of 2D meta-differentiator can be conservatively estimated as approximately 0.75λ.

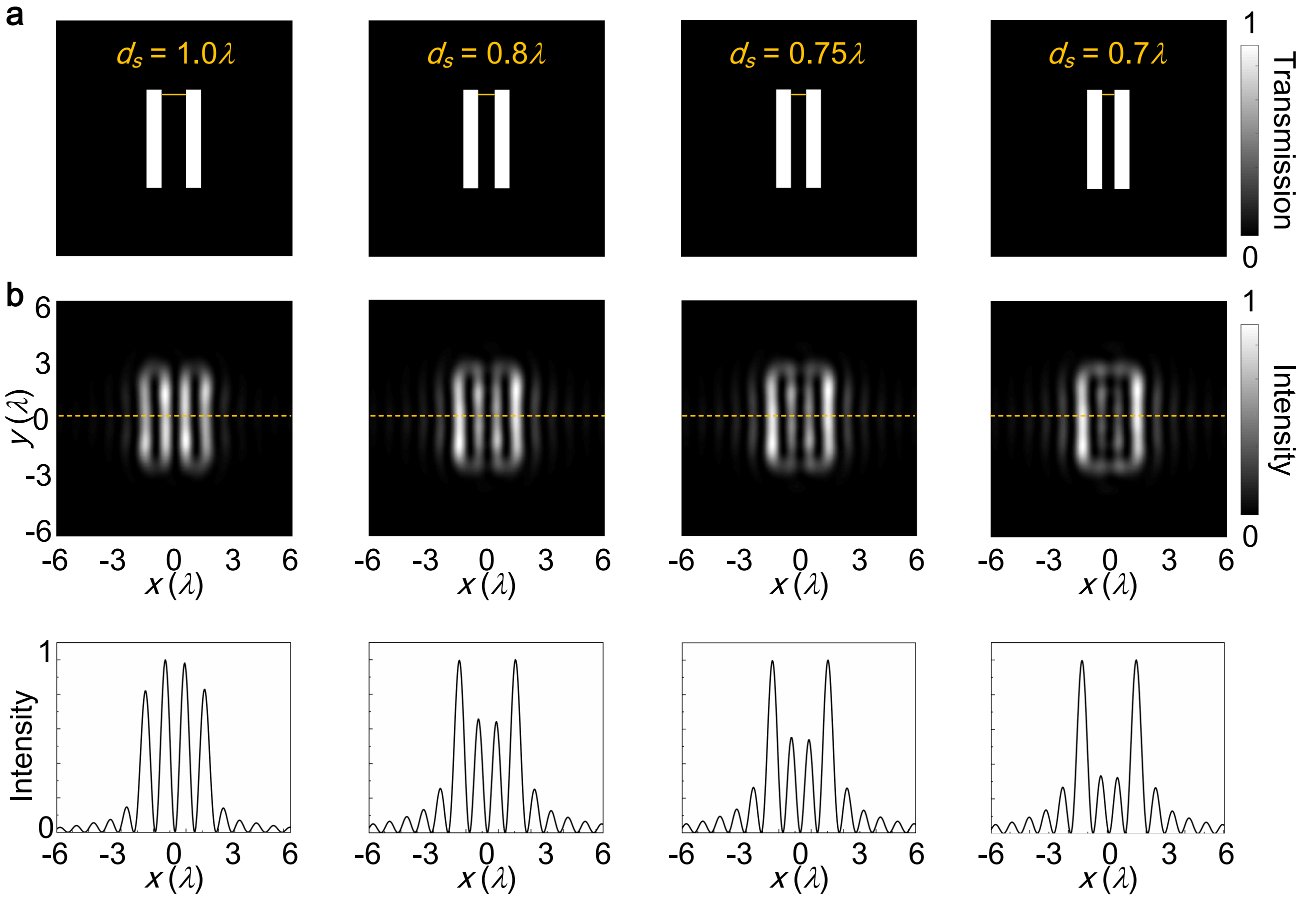

Supplementary Figure 8. **Critical resolution of edge-enhanced images. a** Amplitude objects including two identical rectangles with the dimensions of length 5λ, width 0.75λ, and the separation d_s ranging between λ, 0.8λ, 0.75λ and 0.7λ. **b** Upper panel: calculated acoustic intensity profiles at the imaging plane. Lower panel: line-scanning intensity profiles extracted from the yellow dashed lines indicated in upper panel.

Supplementary Note 10. Frequency response of acoustic meta-differentiator

In the imaging system, the input object is positioned at $z = -f_1$, propagates through the acoustic meta-differentiator located at $z = 0$, and ultimately arrives at the output imaging plane situated at $z = f_2$. The meta-differentiator operates at a designated frequency of 1 MHz, and possesses a specific design with a radius of 30λ (45 mm) and a focal distance of $f = 40\lambda$ (60 mm). An amplitude object, representing the numeral 2, exhibits dimensions with a height of 6λ (9 mm) and a width of 3.75λ (5.625 mm), incorporating a narrow slit measuring 0.75λ (1.125 mm) in width. When the operating frequency is adjusted to 0.9 MHz, the resulting acoustic intensity distribution transmitted through the meta-differentiator along the propagation path is illustrated in the left panel of Supplementary Fig. **9**a. It is discernible that the imaging plane shifts to $f_2 = 67.5$ mm. Furthermore, the acoustic intensity and phase distributions, extracted from the imaging plane, are depicted in the top right and bottom right panels of Supplementary Fig. **9**a, illustrating that all edges of the numeral 2 are prominently highlighted with nearly uniform intensity. Similarly, we explore the scenario involving an operating frequency of 1.0 MHz, and the corresponding acoustic intensity and phase profiles are presented in Supplementary Fig. **9**b. In this case, the imaging plane shifts to $f_2 = 60$ mm, and the intensity profile at the imaging plane accurately delineates all boundary information of the numeral 2. Supplementary Figure **9**c showcases the acoustic intensity and phase distributions when the acoustic meta-differentiator operates at 1.1 MHz. Here, the imaging plane f_2 relocates to 54 mm, and the numeral 2 becomes distinctly discernible with all boundaries enhanced by uniform intensity. It is pertinent to note that the size of output image increases with the frequency, adhering to a similar imaging rule as optical lenses. The imaging plane of 2D meta-differentiator dynamically shifts with changes in the excitation frequency, which bears resemblance to the characteristics observed in Fresnel zone plates [9, 10]. Therefore, the proposed meta-differentiator may not be optimally suited for broadband signal processing, as its design optimization is rooted in single-frequency operation with a fixed imaging distance. However, it is crucial to emphasize that despite this inherent limitation, the meta-differentiator consistently excels in its core capability of edge detection, maintaining its effectiveness across a spectrum of frequency adjustments. Furthermore, the phenomenon of alterations in the imaging plane as a function of operational frequency exhibits potential utility in the context of imaging objects at different depths.

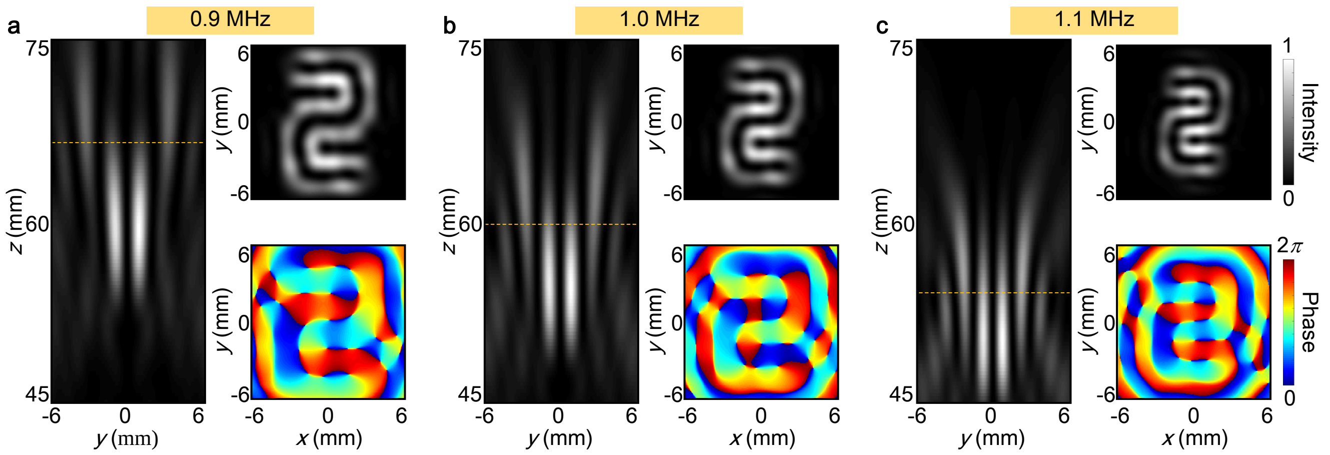

Supplementary Figure 9. **Imaging performance of acoustic meta-differentiator at various excitation frequencies.** **a** Acoustic intensity distribution after transmitting through the meta-differentiator along the propagation direction at the operating frequency of 0.9 MHz (left panel). Acoustic intensity (top right panel) and phase (bottom right panel) distributions extracted from the imaging plane $z = 67.5$ mm as denoted by the yellow dashed line. **b-c** Same as (**a**), but for the acoustic field at the operating frequencies of 1.0 MHz and 1.1 MHz, respectively.

Supplementary Note 11. Edge detection of objects placed at different positions

Here we delve into further investigations regarding the performance of acoustic meta-differentiator with the objects positioned at different distances. In the imaging system, the input image is initially placed at $z = -f_1$, then passes through the acoustic meta-differentiator situated at $z = 0$, culminating in the output image located at the plane $z = f_2$. The acoustic meta-differentiator is engineered to operate at a frequency of 1 MHz, boasting a specific design with a radius of 30λ (45 mm) and a focal distance of $f = 40\lambda$ (60 mm). The amplitude object, symbolizing the numeral 2, possesses dimensions with a height of 6λ and a width of 3.75λ, and includes a slit measuring 0.75λ in width. When the object plane is shifted closer to the acoustic meta-differentiator, specifically at $f_1 = 36\lambda$, the resulting acoustic intensity distribution after transmitting through the meta-differentiator along the propagation path is depicted in the left panel of Supplementary Fig. **10**a. In this scenario, the imaging plane is displaced to $f_2 = 45\lambda$, as determined by the lens imaging equation $2/f = 1/f_1 + 1/f_2$. Furthermore, the top-right and bottom-right panels in Supplementary Fig. **10**a exhibit the acoustic intensity and phase distributions extracted from the imaging plane, clearly illustrating that all edges of the numeral 2 are prominently highlighted with nearly uniform intensity. We also explore the case where the object plane is precisely positioned at $f_1 = 40\lambda$, aligning with the focal position of the acoustic meta-differentiator. The corresponding acoustic intensity and phase profiles are presented in Supplementary Fig. **10**b. In this instance, the imaging plane resides at $f_2 = 40\lambda$, and the intensity profile at the imaging plane accurately captures all boundary information of the object, faithfully representing the shape of the numeral 2. Additionally, we have examined the scenario in which the object plane is situated at a greater distance away from the acoustic meta-differentiator, specifically at $f_1 = 44\lambda$. The corresponding acoustic intensity and phase profiles are presented in Supplementary Fig. **10**c. It is evident that the imaging plane shifts to $f_2 = 36.7\lambda$, and the intensity profile at the imaging plane accurately captures all edge information of the object. Moreover, when the object is moved closer to the acoustic meta-differentiator, the resulting image undergoes proportional enlargement, in accordance with the imaging principles of optical lenses. In conclusion, our proposed meta-differentiator exhibits the capability to effectively detect objects positioned at varying distances, and the resultant enlargement or minification effects can be harnessed to observe minute objects in greater detail.

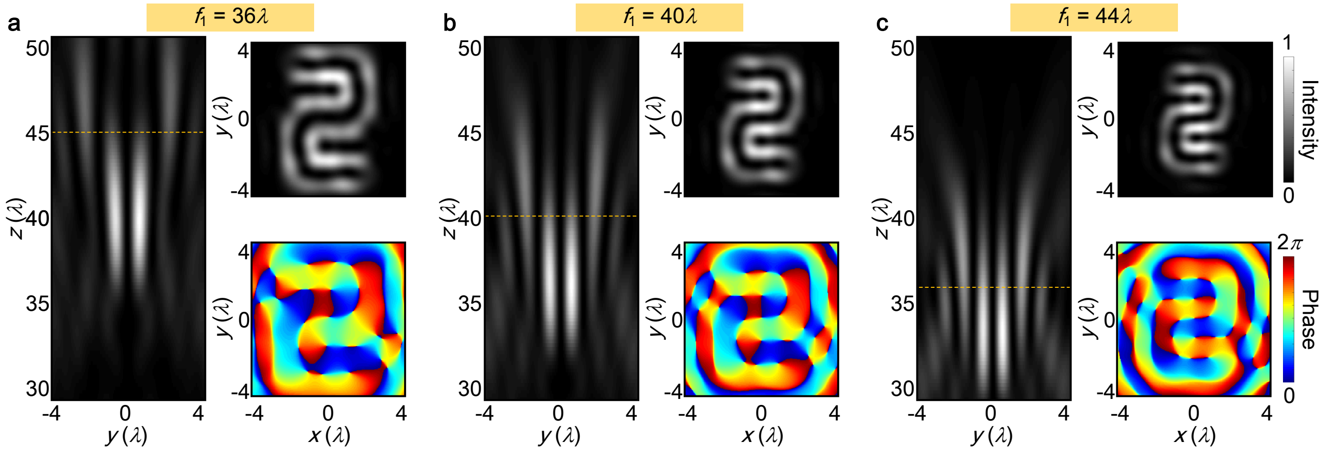

Supplementary Figure 10. Imaging performance of acoustic meta-differentiator with the object placed at various positions. a Acoustic intensity distribution after transmitting through the meta-differentiator along the propagation path with the object placed at $f_1 = 36\lambda$ (left panel). Acoustic intensity (top right panel) and phase (bottom right panel) distributions extracted from the imaging plane at $f_2 = 45\lambda$ as denoted by the yellow dashed line. **b** Equivalent to (**a**), but for the acoustic field with the object and imaging planes residing at $f_1 = 40\lambda$ and $f_2 = 40\lambda$, respectively. **c** Same as (**a**), but for the object and imaging planes located at $f_1 = 44\lambda$ and $f_2 = 36.7\lambda$, respectively.

Supplementary Note 12. Effects of amplitude and phase discretization on 2D spatial differentiation

To replicate the continuous amplitude and phase profiles required for the spatial differentiator experimentally is challenging. Therefore, we discretize these profiles and investigate how discretization affects the behavior of 2D spatial differentiation. Specifically, we consider amplitude and phase profiles given by $A = |r|$ and $\varphi = -2k(\sqrt{r^2+f^2}-f)+\theta$, respectively. Supplementary Figure **11**a presents a phase profile split into 2 steps of 0 and π, with the amplitude profile discretized into 16 steps (referred to as 2PM-16AM). The scale bar represents a length of 15λ. At the input plane, an amplitude object of the numeral 2, measuring 6λ in height, 3.75λ in width, and featuring a slit of 0.75λ, is placed. The calculated acoustic intensity distribution at the output plane is shown in the lower panel. It can be observed that all boundaries of the numeral 2 are clearly visible, albeit with low intensities. This demonstrates that 2PM-16AM enables 2D isotropic spatial differentiation but with reduced efficiency. Next, we examine a phase profile divided into 4 steps of 0, $\pi/4$, $2\pi/4$ and $3\pi/4$ (referred to as 4PM-16AM), as depicted in Supplementary Fig. **11**b. From the output image, it is evident that all outlines of the numeral 2 are enhanced with improved intensity, confirming that 4PM-16AM achieves more efficient 2D edge-enhanced imaging. We also investigate the phase profiles with 8 steps (8PM-16AM) and 16 steps (16PM-16AM), as shown in Supplementary Figs. **11**c and **11**d. In both cases, isotropic edge enhancements are clearly observed, with a significant improvement in acoustic intensity as the number of phase steps increases. Furthermore, we explore the influence of amplitude discretization on the 2D spatial differentiator in Supplementary Figs. **11**e-h. We find that using 16 amplitude steps (referred to as 16AM) is sufficient to ensure high-resolution imaging efficiency. Considering the trade-off between fabrication complexity and energy efficiency, we select the 2PM-16AM design for preparing the experimental sample.

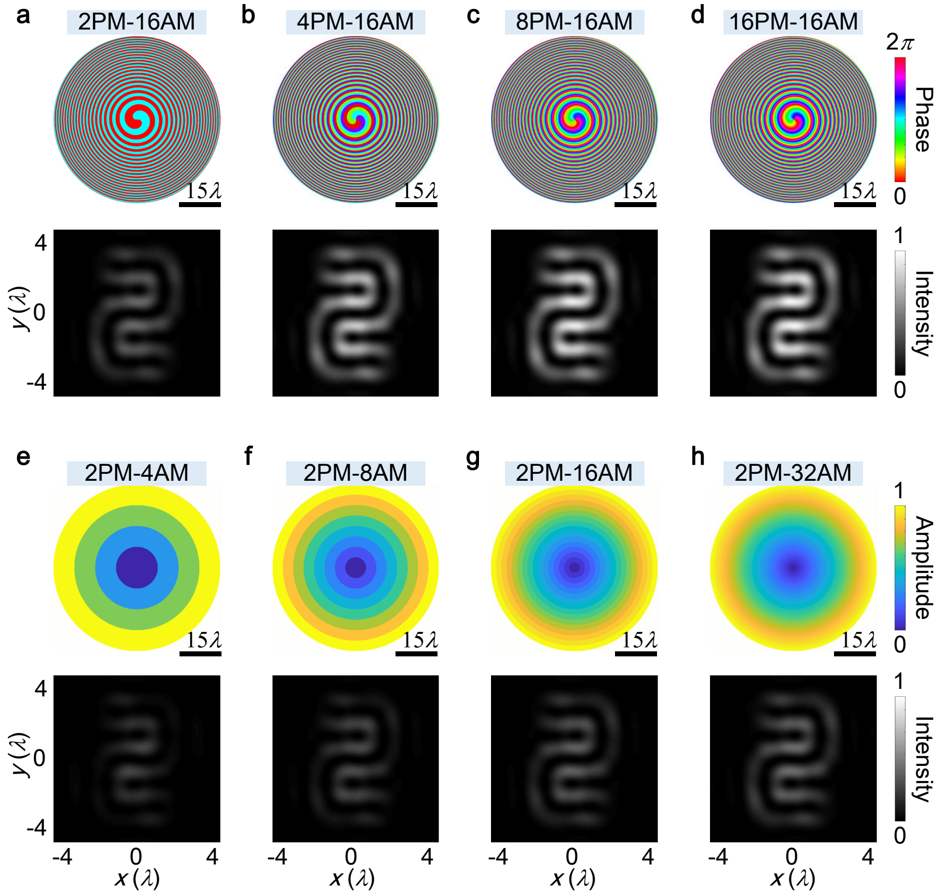

Supplementary Figure 11. **Effects of amplitude and phase discretization on 2D spatial differentiation. a-d** Upper panel: phase profile discreted into 2, 4, 8, and 16 steps, with the amplitude profile discretized into 16 steps (referred to as 2PM-16AM, 4PM-16AM, 8PM-16AM, and 16PM-16AM). Lower panel: calculated acoustic intensity distribution at the output plane. **e-h** Same as (**a**)-(**d**), but for the amplitude profile discreted into 4, 8, 16, and 32 steps, with the phase profile discretized into 2 steps (referred to as 2PM-4AM, 2PM-8AM, 2PM-16AM, and 2PM-32AM).

Supplementary Note 13. Cross-sectional view of acoustic meta-differentiator

We have presented the cross-sectional views of acoustic differentiator, with both the phase and amplitude meta-gratings illustrated in Supplementary Figs. **12**a and **12**b, respectively. The distributions of thickness and width parameters are also plotted in the corresponding panels. For the phase meta-grating, the discrete binary phase profile with a π phase difference is represented by variations in resin height (h), with thicknesses of 0.45 mm and 2.25 mm (depicted in yellow). To reduce wave interactions between neighboring resin units, a stainless steel sheet with a height of 3.75 mm and a thickness of 200 μm (depicted in gray) is inserted between adjacent resin units. Regarding the amplitude meta-grating, the cross-sectional view reveals a stainless steel plate engraved with 14 annular slits, exhibiting varying widths (w) ranging from 0.2 mm to 2.8 mm with an interval of 0.2 mm along the radial direction.

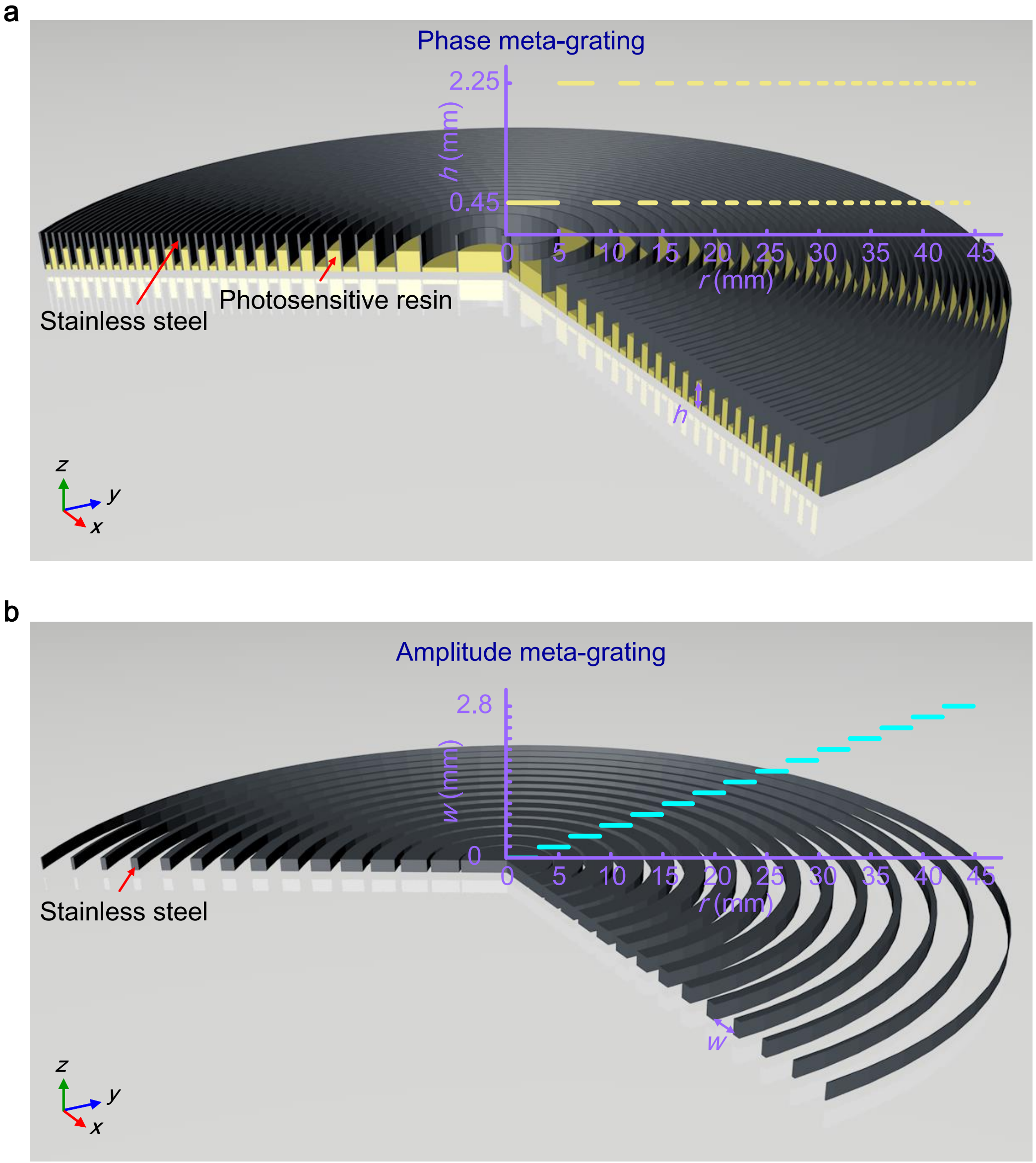

Supplementary Figure 12. Cross-sectional views of both (**a**) phase and (**b**) amplitude meta-gratings in an acoustic differentiator, with a plot of the thickness and width parameters that are implemented.

Supplementary Note 14. Experimental results for amplitude object in different size

Supplementary Figure **13**a displays an amplitude object in the shape of a small numeral 2 with a height of 4λ, a width of 2.5λ, and a slit of 0.5λ, where λ is 1.5 mm. In this object, the white regions represent full transmission of acoustic waves, while the black areas indicate no acoustic waves. Supplementary Figures **13**b and **13**c present the calculated and simulated acoustic intensity and phase profiles at the output plane, respectively. It can be observed that the edges of numeral 2 are enhanced isotropically with an almost uniform intensity distribution. Next, we fabricate a stainless steel object representing a small numeral 2, as shown in Supplementary Fig. **13**d. The corresponding intensity and phase distributions measured at the output plane are given in Supplementary Fig. **13**e, which are consistent with the simulated results. To further validate our findings, Supplementary Fig. **13**f shows the intensity profiles along the vertical direction, comparing the theoretical, numerical, and experimental results. It is evident that the edge-enhanced parts agree well with each other.

Moving on to larger amplitude objects, Supplementary Fig. **13**g exhibits an amplitude object in the shape of of a large numeral 2 with a height of 8λ, a width of 5λ, and a slit of 1λ. The corresponding calculated and simulated results are presented in Supplementary Figs. **13**h and **13**i, outlining all boundaries of the numeral 2. These results demonstrate the excellent ability of our spatial differentiator in enhancing edges. To validate the findings for larger amplitude objects experimentally, we fabricate a stainless steel object representing a large numeral 2, as shown in Supplementary Fig. **13**j. The experimental results at the output plane are displayed in Supplementary Fig. **13**k. Additionally, Supplementary Fig. **13**l provides the intensity profiles along the vertical axis, comparing the theoretical, numerical, and experimental results. It is observed that the measured results align closely with the simulations, with slight deviations attributed to fabrication errors in the experimental sample. Hence, our 2D spatial differentiator proves effective in enhancing edge details for amplitude objects of varying sizes.

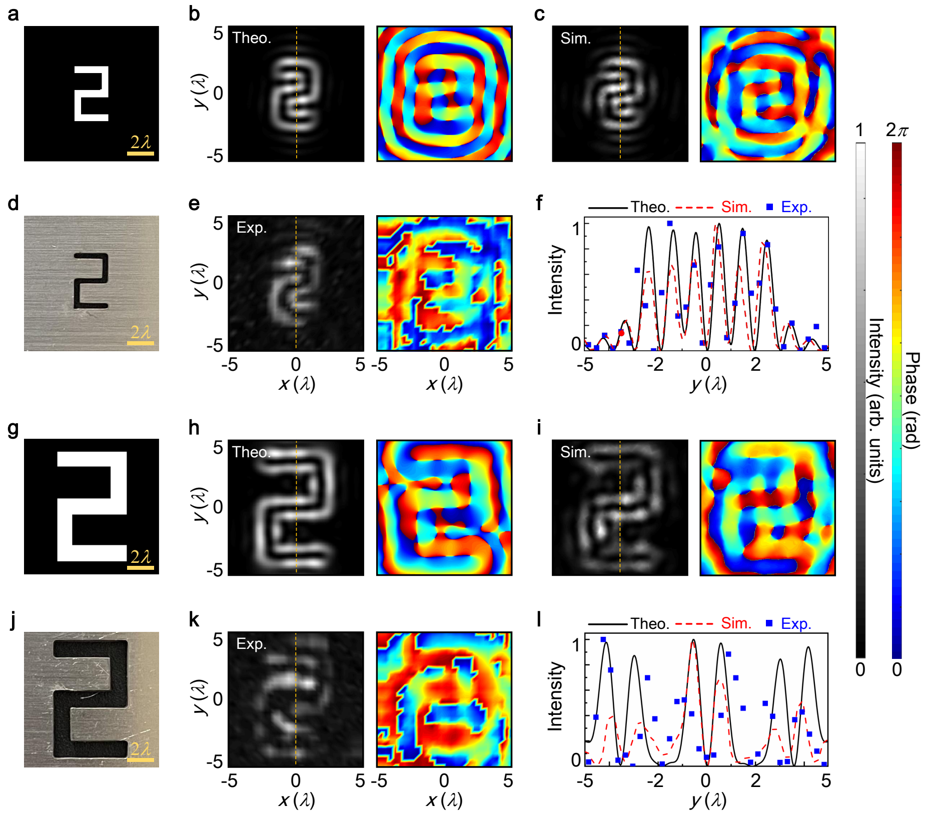

Supplementary Figure 13. **Edge-enhanced imaging of amplitude objects in different sizes. a** An amplitude object in the shape of a small numeral 2 with a height of 4λ, a width of 2.5λ, and a slit of 0.5λ. **b** Calculated and (**c**) simulated intensity and phase profiles at the output plane. **d** Fabricated amplitude object of the small numeral 2 made from stainless steel. **e** Measured intensity and phase profiles at the output plane. **f** Intensity profiles along the vertical direction, comparing the theoretical, numerical, and experimental results. The edge-enhanced parts show good agreement across all three sets of data. **g-l** Same as (**a**)-(**f**), but for the amplitude object in the shape of a large numeral 2 with a height of 8λ, a width of 5λ, and a slit of 1λ.

Supplementary Note 15. Experimental results for amplitude object in different shape

Supplementary Figure **14**a displays an amplitude object in the shape of a numeral 3, featuring a height of 6λ, a width of 3.75λ, and a slit of 0.75λ. Supplementary Figures **14**b and **14**c present the calculated and simulated acoustic intensity and phase profiles, respectively, at the output plane. All boundaries of the numeral 3 exhibit enhanced isotropic uniform intensity distribution. To further demonstrate this effect, we fabricated an amplitude object of the numeral 3 (Supplementary Fig. **14**d) using the stainless steel plate with a thickness of 1.2 mm. The corresponding intensity and phase distributions measured at the output plane are depicted in Supplementary Fig. **14**e, which show good agreement with the simulated results. Additionally, Supplementary Fig. **14**f illustrates the intensity profiles along the vertical direction, comparing the theoretical, numerical, and experimental results, thus confirming the high efficiency of the 2D spatial differentiator in edge detection of various object shapes.

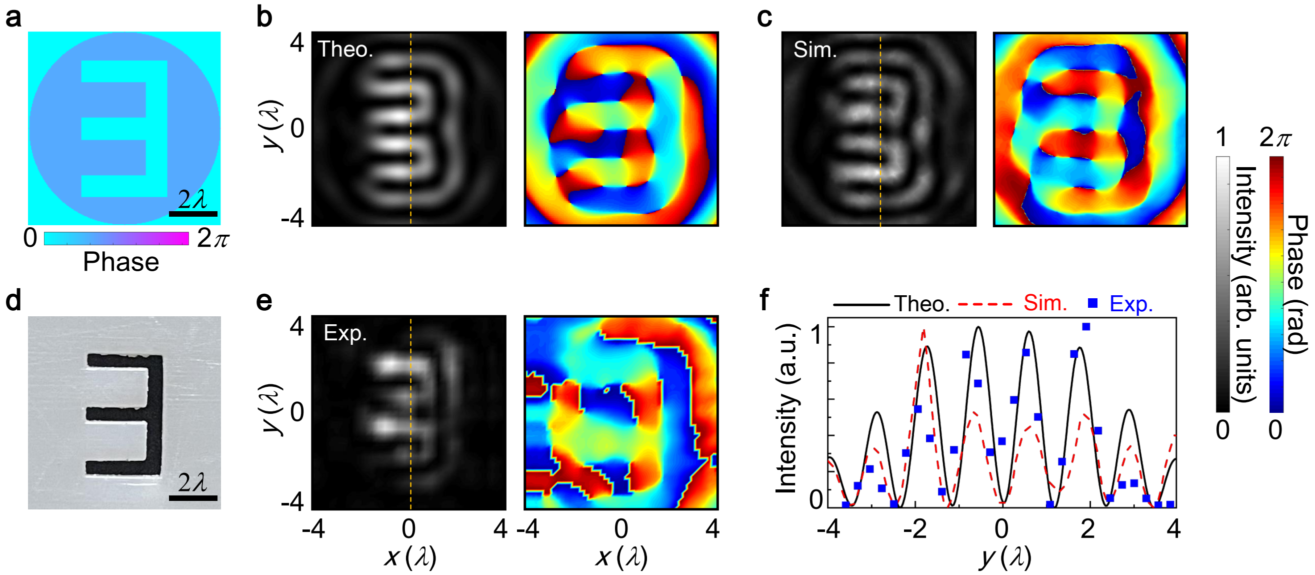

Supplementary Figure 14. **Edge-enhanced imaging for amplitude object in different shape.** **a** An amplitude object in the shape of numeral 3 with a height of 6λ, a width of 3.75λ, and a slit of 0.75λ. **b** Calculated and (**c**) simulated intensity and phase profiles at the output plane. **d** Photograph of the amplitude object of numeral 3 fabricated from stainless steel. **e** Measured intensity and phase profiles at the output plane. **f** Intensity profiles along the vertical direction, comparing the theoretical, numerical, and experimental results.

Supplementary Note 16. Demonstration of Reflective-mode Meta-differentiator

In addition to the transmission-mode meta-differentiator in the manuscript, we further introduce a reflective-mode acoustic meta-differentiator as illustrated in Supplementary Fig. **15**a. In this configuration, ultrasonic waves pass through an object situated at the plane $z = -f$, subsequently impinge upon the reflected-mode meta-differentiator positioned at $z = 0$, and ultimately undergo reflection, culminating in the formation of an image with enhanced edge delineation at the incident plane $z = -f$. The amplitude and phase meta-gratings applied to the reflective-mode meta-differentiator are depicted in Supplementary Figs. **15**b and **15**c, respectively. Differing from the transmission-mode setup, the resin height distributions on the reflected-mode phase grating are halved due to the doubled reflected acoustic distance, and are set as 0.15λ and 0.75λ, respectively, to ensure π differences between two reflected phase steps. Meanwhile, the reflected-mode amplitude modulator is entirely inverted compared to its transmission-mode counterpart, with sound transmittivity decreasing with radial distance to increase reflectivity along the radial direction. In Supplementary Fig. **15**d, we introduce an amplitude object featuring a small circular hole with a radius of 0.5λ at the input plane $z = -f$. This object serves to confirm the PSF of the reflective-mode meta-differentiator in the imaging system. Supplementary Figure **15**e presents simulated acoustic intensity and phase distributions of the reflected field at the image plane $z = -f$. It should be emphasized that the incident waves are continuous rather than impulse waves, thus the reflected field is obtained by subtracting the incident waves from the total field. It is observed that the PSF of reflective-mode meta-differentiator manifests as a high-intensity doughnut pattern accompanied by a spiral phase carrying a 1st-order topological charge, akin to that of the transmission-mode counterpart. Furthermore, we examine the case of an amplitude object shaped like the numeral 2, with dimensions of 6λ in height, 3.75λ in width, and a slit measuring 0.75λ, as illustrated in Supplementary Fig. **15**f. The corresponding acoustic intensity and phase profiles of reflected fields are depicted in Supplementary Fig. **15**g, wherein all edges of the numeral 2 are prominently highlighted with isotropic intensity distributions. Hence, the reflective-mode meta-differentiator demonstrates outstanding performance in edge extraction capabilities as well.

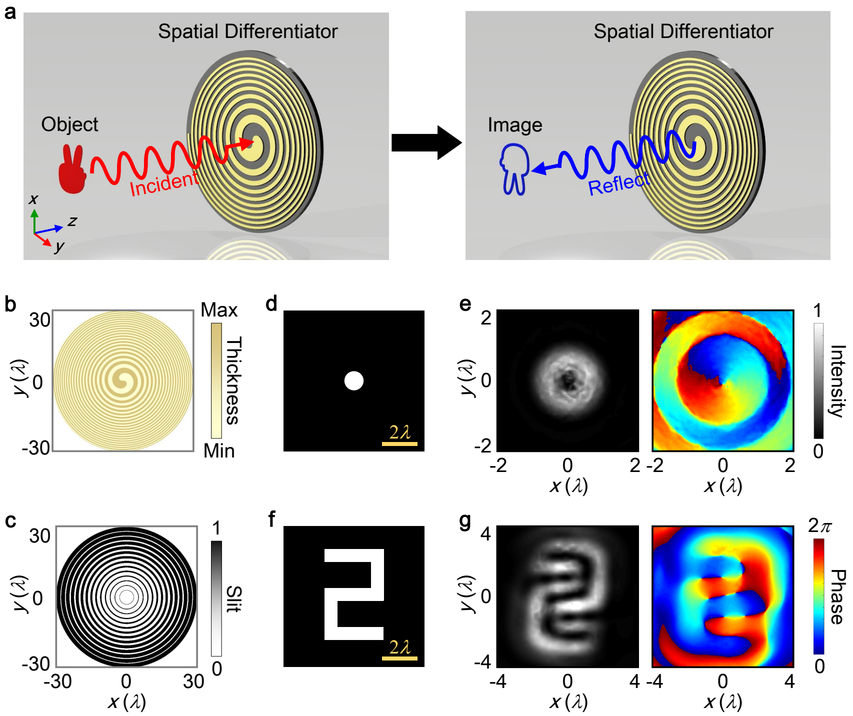

Supplementary Figure 15. **Reflective-mode meta-differentiator for edge enhancement of amplitude objects. a** Schematic illustration of employing a reflective-mode meta-differentiator in the edge-enhanced imaging system. **b** Discrete phase modulations on the reflective meta-differentiator mapped to the distribution of resin thickness. **c** Discrete amplitude modulations represented by the distributions of slit width. **d** An amplitude object featuring a small circular hole with a radius of 0.5λ at the input plane $z = -f$. **e** Simulated intensity and phase distributions of the reflected acoustic field at the image plane $z = -f$. **f**-**g** Same as (**d**)-(**e**), but featuring an amplitude object in shape of the hollow numeral 2 with dimensions of 6λ in height, 3.75λ in width, and a slit measuring 0.75λ.

Supplementary Note 17. Reflective imaging system utilizing transmission-mode meta-differentiator

We also propose a reflective imaging methodology with the utilization of transmission-mode meta-differentiator to process backscattering waves, which is specifically designed for pragmatic deployment in clinical settings (refer to Supplementary Fig. **16**a for visual representation). In the simulation, the background sound field emits Gaussian acoustic waves with the radius of 4λ directed towards an object situated at the plane $z = f$. The subsequently reflected scattering waves then traverse through the transmission-mode meta-differentiator positioned at $z = 0$, and ultimately coalesce to form an image characterized by edge enhancements at imaging plane $z = -f$. An amplitude object featuring a small circle with a radius of 0.5λ is placed at the object plane $z = f$, as depicted in Supplementary Fig. **16**b. The majority of acoustic waves bypass the small circular object, while a fraction of them are reflected to form an image of circle. After post-meta-differentiator processing, the simulated intensity and phase distributions of the acoustic field at image plane $z = -f$ are presented in Supplementary Fig. **16**c. Notably, the scattered PSF of the meta-differentiator exhibits a high-intensity doughnut pattern accompanied by a spiral phase carrying a 1st-order topological charge, consistent with the findings detailed in the manuscript. Furthermore, we consider the case of an amplitude object shaped like the numeral 2, with dimensions of 6λ in height, 3.75λ in width, and a line thickness measuring 0.75λ, as depicted in Supplementary Fig. **16**d. The corresponding intensity and phase profiles for the scattered waves processed by the meta-differentiator are displayed in Supplementary Fig. **16**e. It is observed that all edges of the numeral 2 are accentuated with isotropic intensity distributions, affirming the efficacy of transmission-mode meta-differentiator in edge detection of scattered waves. Conclusively, our proposed meta-differentiator serves as a robust image processing tool for the edge detection of both transmitted and scattered fields. A subsequent consideration pertains to the transition from continuous waves to pulse waves within the reflective-mode imaging scheme, a shift with promising implications for the practical utilization of the proposed meta-differentiator in clinical settings for medical diagnosis.

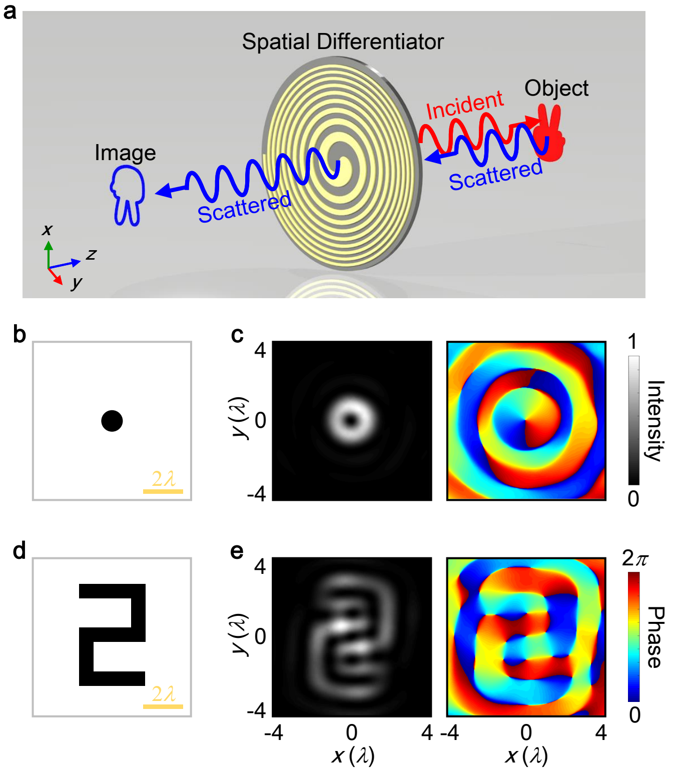

Supplementary Figure 16. **Reflective edge-enhanced imaging system employing a transmission-mode meta-differentiator.** **a** Schematic of reflective edge-enhanced imaging with the employment of a transmission-mode meta-differentiator within the imaging system. **b** Amplitude object in the shape of a small circle with a radius of 0.5λ. **c** Simulated intensity and phase distributions of the acoustic field at the image plane. **d-e** Equivalent to (**b**)-(**c**), but featuring an amplitude object in the form of the numeral 2 with dimensions of 6λ in height, 3.75λ in width, and a line thickness measuring 0.75λ.

Supplementary Note 18. Imaging of phase objects with different phase gradients

Supplementary Figure **17**a shows the phase object in the shape of numeral 2 with a height of 6λ, a width of 3.75λ, and a slit of 0.75λ. The incident Gaussian plane wave has a width of 8λ and undergoes a phase advance of a $\pi/4$. This phase advance is employed to generate a phase difference of $\pi/4$ between the phase object and the ambient medium. The simulated acoustic intensity profile at the output plane is presented in the lower panel. It is observed that not only are all boundaries of numeral 2 isotropically enhanced due to the abrupt phase variations, but the edges of incident Gaussian waves are also highlighted because of the amplitude jumps. When the phase shift is adjusted to $\pi/2$ in Supplementary Fig. **17**b, the acoustic intensity along the sketches of numeral 2 is improved, while that of the incident field is decreased. Supplementary Figures **17**c and **17**d illustrate the Gaussian waves with phase jumps of $3\pi/4$ and π, respectively. In these cases, the contour lines between different phases become more pronounced, and the edges of the incident field are clearly visible. Hence, it is possible to obtain isotropic edge-enhanced imaging of phase objects with slow phase variations.

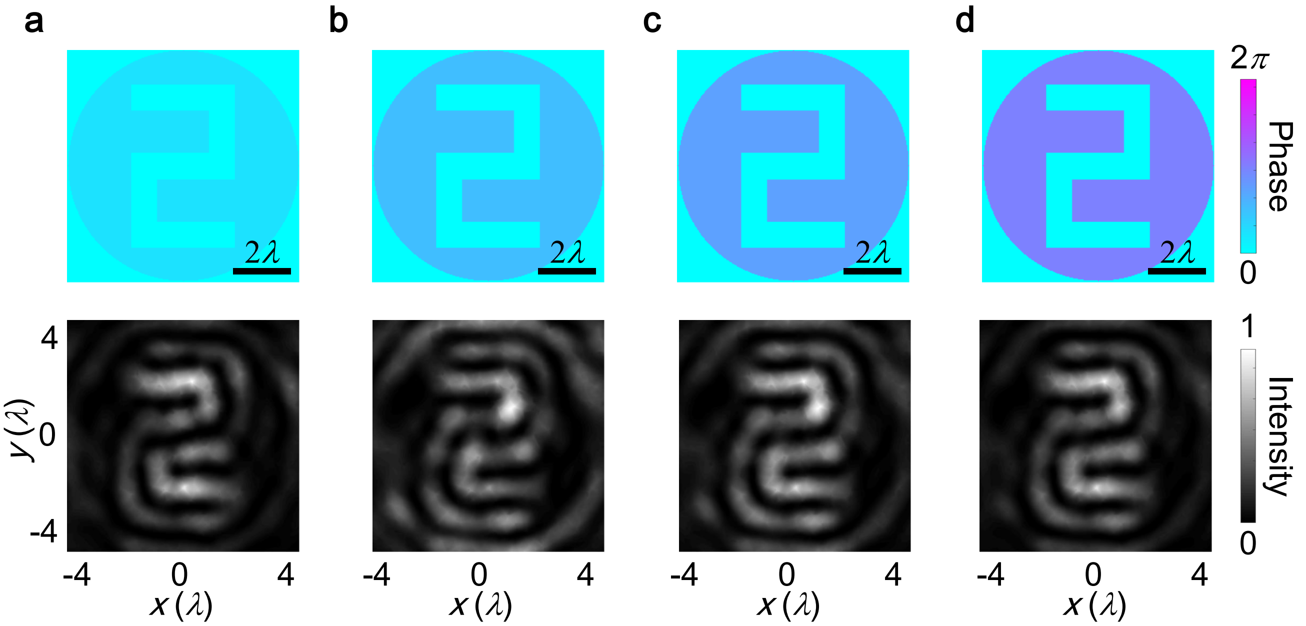

Supplementary Figure 17. **Imaging of phase objects with different phase gradients. a-d** Upper panel: phase objects illuminated by Gaussian acoustic waves with different phase gradients of $\pi/4$, $2\pi/4$, $3\pi/4$, π. Lower panel: simulated acoustic intensity distributions at the output plane.

Supplementary Note 19. Control experiments on object imaging with and without acoustic meta-differentiator

Supplementary Figure **18**a illustrates an amplitude object engraved into a steel plate, featuring a hollow pattern resembling the numeral 2. This object possesses dimensions of 6λ in height, 3.75λ in width, and a slit measuring 0.75λ. Here, the ultrasonic transducer is operated at 1 MHz ($\lambda = 1.5$ mm). In Supplementary Fig. **18**b, we present the simulated and measured acoustic intensity distributions at the imaging plane, which is located $z = 80\lambda$ away from the amplitude object. This is done without using the proposed meta-differentiator. It is observed that the configuration of the numeral 2, which has an extremely weak intensity, is hardly discernible due to sound field diffraction. As comparison, Supplementary Fig. **18**c displays the simulated and measured acoustic intensity distributions that have been processed by the meta-differentiator. The meta-differentiator successfully extracts and highlights all the boundaries of the numeral 2 with a uniform intensity, underscoring its role as an image edge extractor.

In addition, we examine a phase object in the shape of numeral 2, both with and without the use of the meta-differentiator. The phase object is prepared from a circular resin plate with the diameter of 90 mm, and a hollow numeral 2 with the same dimensions as the amplitude object is engraved at its center, as depicted in Supplementary Fig. **18**d. The resin plate has a thickness of 1.2 mm, which ensures complete transmission of ultrasonic waves and introduces a phase delay of $2\pi/3$ compared to the hollow region. The ultrasonic transducer emits a Gaussian plane wave with a width of 12.7 mm. In Supplementary Fig. **18**e, we present the simulated and measured acoustic fields at the plane located $z = 80\lambda$ away from the phase object without being processed by the meta-differentiator. It is evident that no information regarding the numeral 2 is revealed in the far-field acoustic field distribution. In contrast, Supplementary Fig. **18**f showcases the simulated and measured intensity profiles at the output plane with the application of acoustic meta-differentiator. It reveals an isotropic enhancement of intensity distribution along all edges of the numeral 2.

For further validation, we examine a complementary physical numeral 2 made of resin material, featuring a thickness of 1.2 mm and a phase advance of $2\pi/3$ relative to the ambient medium (Supplementary Fig. **18**g). The acoustic intensity profiles of the phase objects, with and without the assistance of the meta-differentiator, are illustrated in Supplementary Figs. **18**h and **18**i, respectively. Without the meta-differentiator, it is challenging to distinguish the phase object from the surrounding medium. However, with the use of the acoustic meta-differentiator, all edges of the numeral 2 exhibit significant enhancements for easy recognition. Consequently, the meta-differentiator facilitates easy identification of both amplitude and phase object contours in the far field ($80\lambda \gg$critical distance $R^2/\lambda = 16\lambda$ with R being the radius of transducer), and aids in distinguishing objects with phase differences from the ambient background.

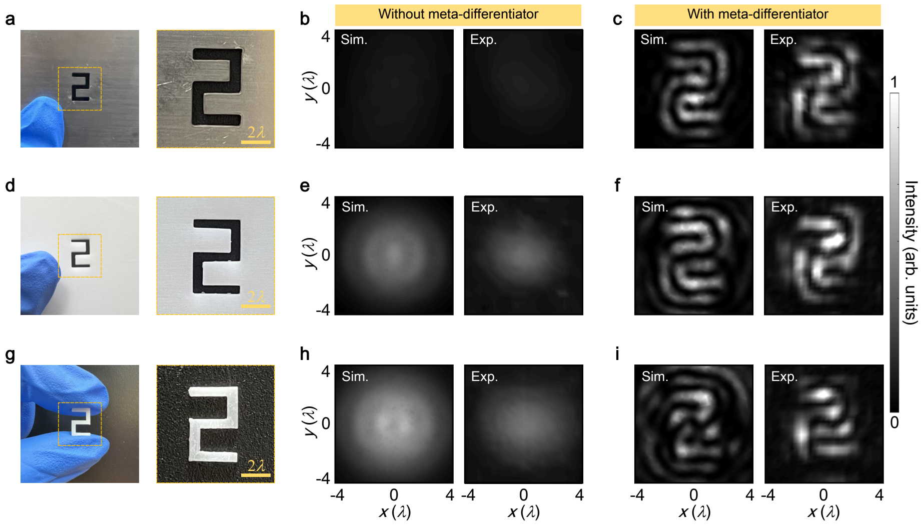

Supplementary Figure 18. **Control experiments on target object imaging in the presence and absence of acoustic meta-differentiator. a** An amplitude object crafted from a steel plate featuring a hollow pattern of the numeral 2. Simulated and measured acoustic intensity distributions at the imaging plane $z = 80\lambda$ away from the amplitude object (**b**) without and (**c**) with the employment of acoustic meta-differentiator. **d-f** Analogous to (**a**)-(**c**), but involving a phase object constructed from a resin plate with a hollow pattern of the numeral 2 at its center. **g-i** Equivalent to (**d**)-(**f**), but featuring a phase object with the complementary physical numeral 2 made of resin material.

Supplementary Note 20. Isotropic edge-enhanced imaging of complex phase objects

We also examine the effectiveness of the spatial differentiator in detecting edges of complex phase objects. The upper panel of Supplementary Figs. **19**a-d displays various phase objects, including numeral 0, 1, 3, and 4. In these images, cyan regions indicate phase uniformity, while purple areas represent the presence of Gaussian acoustic fields with π phase differences. The lower panel showcases the corresponding calculated acoustic intensity distributions at the output plane. Supplementary Figures **19**e-h exhibit additional phase objects featuring alphabet N, J, U, and S (upper panel), along with the respective output images after being processed by the spatial differentiator (lower panel). Notably, all boundaries of the phase objects are distinctly highlighted, confirming the spatial differentiator's excellent performance in edge-enhanced imaging of complex phase objects.

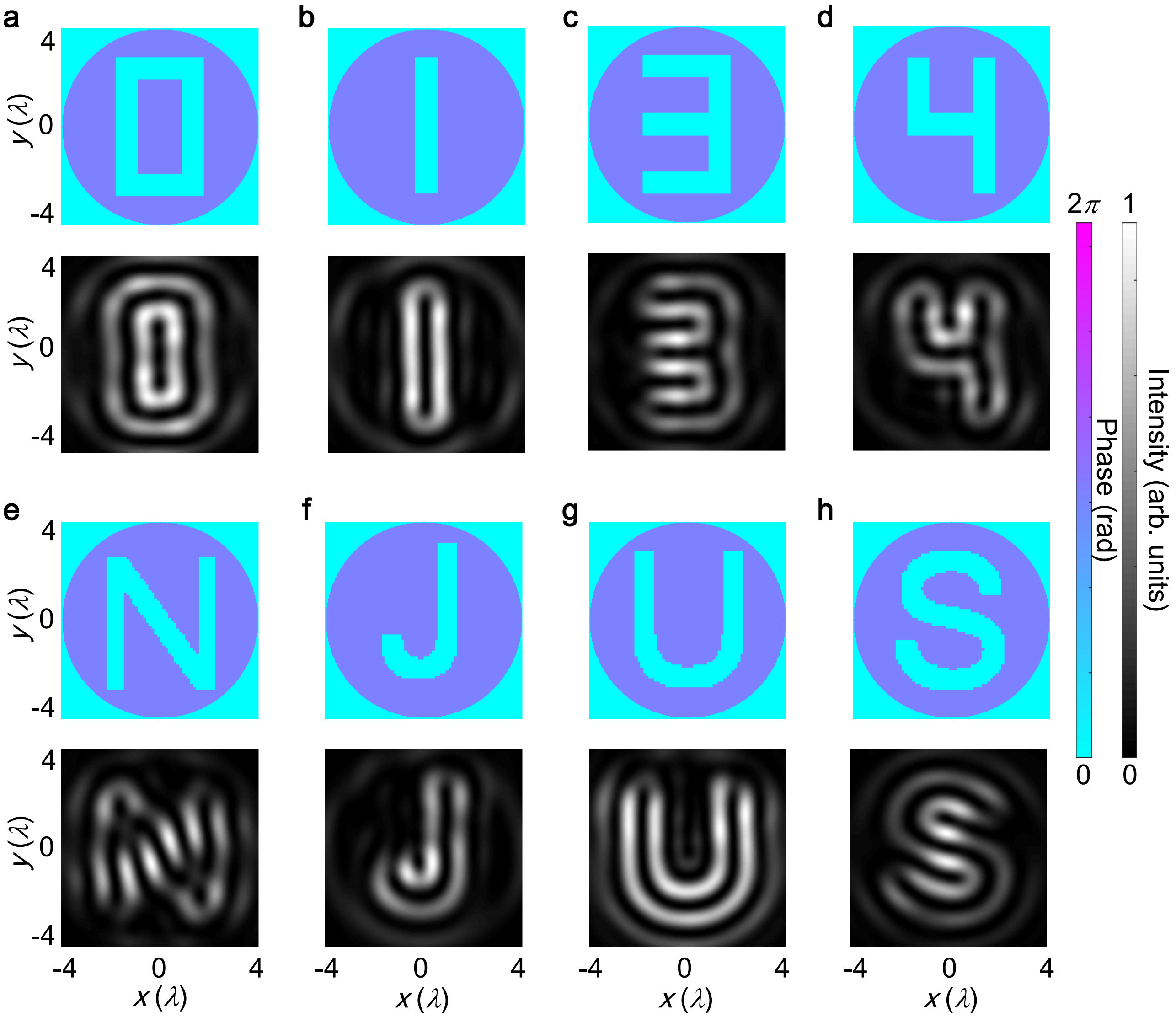

Supplementary Figure 19. **Isotropic edge-enhanced imaging of complex phase objects. a-d** Upper panel: phase objects with different shapes of numeral 0, 1, 3, and 4, which are illuminated by Gaussian acoustic waves with a phase difference of π. Lower panel: calculated acoustic intensity distributions at the output plane. **e-h** Same as (**a**)-(**d**), but for the phase objects in the shapes of alphabet N, J, U, and S.

Supplementary Note 21. Experimental results for phase object in different shape

Supplementary Figure **20**a illustrates an ideal phase object in the shape of numeral 3, with a height of 6λ, a width of 3.75λ, and a slit of 0.75λ, where λ is equal to 1.5 mm. It is illuminated by a Gaussian plane wave with a width of 8λ and a phase advance of $2\pi/3$. The calculated and simulated acoustic intensity and phase profiles at the output plane are shown in Supplementary Figs. **20**b and **20**c, respectively. These results demonstrate that all edges of numeral 3 are enhanced, resulting in a uniform intensity distribution due to the phase difference. Also, the boundaries of the incident acoustic field are outlined as a result of the amplitude jump. Next, we fabricate a phase object of numeral 3, as depicted in Supplementary Fig. **20**d. This phase object is made from resin material with a thickness of 1.2 mm. The corresponding intensity and phase distributions measured at the output plane are presented in Supplementary Fig. **20**e, which are consistent with the simulated results. Supplementary Figure **20**f displays the intensity profiles along the vertical direction, comparing the theoretical, numerical, and experimental results. These plots reveal that the 2D spatial differentiator works effectively in detecting the edges of different phase objects.

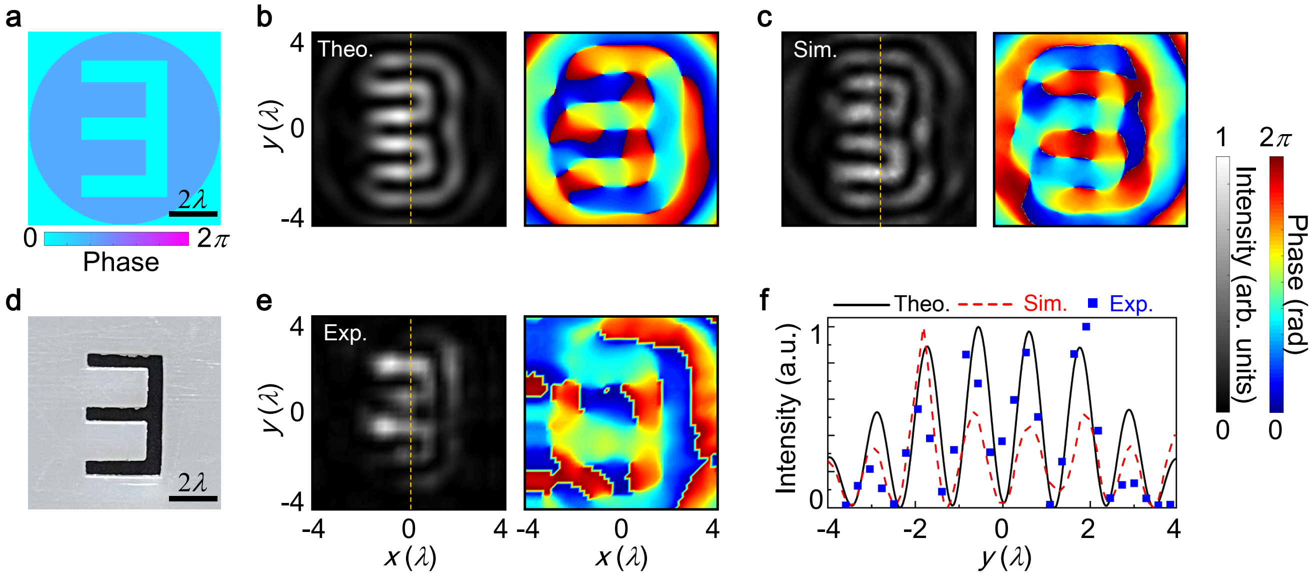

Supplementary Figure 20. **Edge detection of phase object in different shape. a** An ideal phase object in the shape of numeral 3 with a height of 6λ, a width of 3.75λ, and a slit of 0.75λ, illuminated by Gaussian plane wave with a width of 8λ and a phase advance of $2\pi/3$. **b** Calculated and (**c**) simulated intensity and phase profiles at the output plane. **d** Fabricated phase object of numeral 3, created using resin material with a thickness of 1.2 mm. **e** Measured intensity and phase profiles at the output plane. **f** Intensity profiles along the vertical direction, comparing the theoretical, numerical, and experimental results.

Supplementary Note 22. Comparison between focus metasurface and 2D spatial differentiator

Supplementary Figure **21**a displays an amplitude object in the shape of numeral 2 with a height of 6λ, a width of 3.75λ, and a slit of 0.75λ. The scale bar represents a length of 3 mm. Processed by the focus metasurface, the calculated and simulated acoustic intensity profiles of numeral 2 at the output plane are shown in Supplementary Fig. **21**b. It is observed that the whole image of numeral 2 is successfully reconstructed at the output plane. Supplementary Figure **21**c presents the calculated and simulated results of numeral 2 based on the 2D spatial differentiator. All boundaries are enhanced with an isotropic intensity distribution. Therefore, both the focus metasurface and the 2D spatial differentiator demonstrate good performance in processing amplitude objects. In Supplementary Fig. **21**d, a phase object featuring numeral 2 with a height of 6λ, a width of 3.75λ, and a slit of 0.75λ is illuminated by the Gaussian plane wave in 8λ width with a phase advance of $\pi/4$. The corresponding acoustic intensity profiles at the output plane by utilizing focus metasurface and 2D spatial differentiator are presented in Supplementary Figs. **21**e and **21**f, respectively. It is found that the focus metasurface fails to reconstruct the input phase object, while the spatial differentiator is able to discriminate the detailed boundary information of phase object from the ambient medium. Additionally, we discuss the case of a phase object with a phase advance of $\pi/4$ relative to the incident acoustic field in Supplementary Fig. **21**g. The calculated and simulated intensity distributions at the output plane also demonstrate the superiority of the spatial differentiator in edge enhancements of phase objects, as shown in Supplementary Figs. **21**h and **21**i. Compared with the conventional imaging system based on the focus metasurface, the proposed 2D spatial differentiator excels in presenting more detailed information of phase objects with slight phase variations.

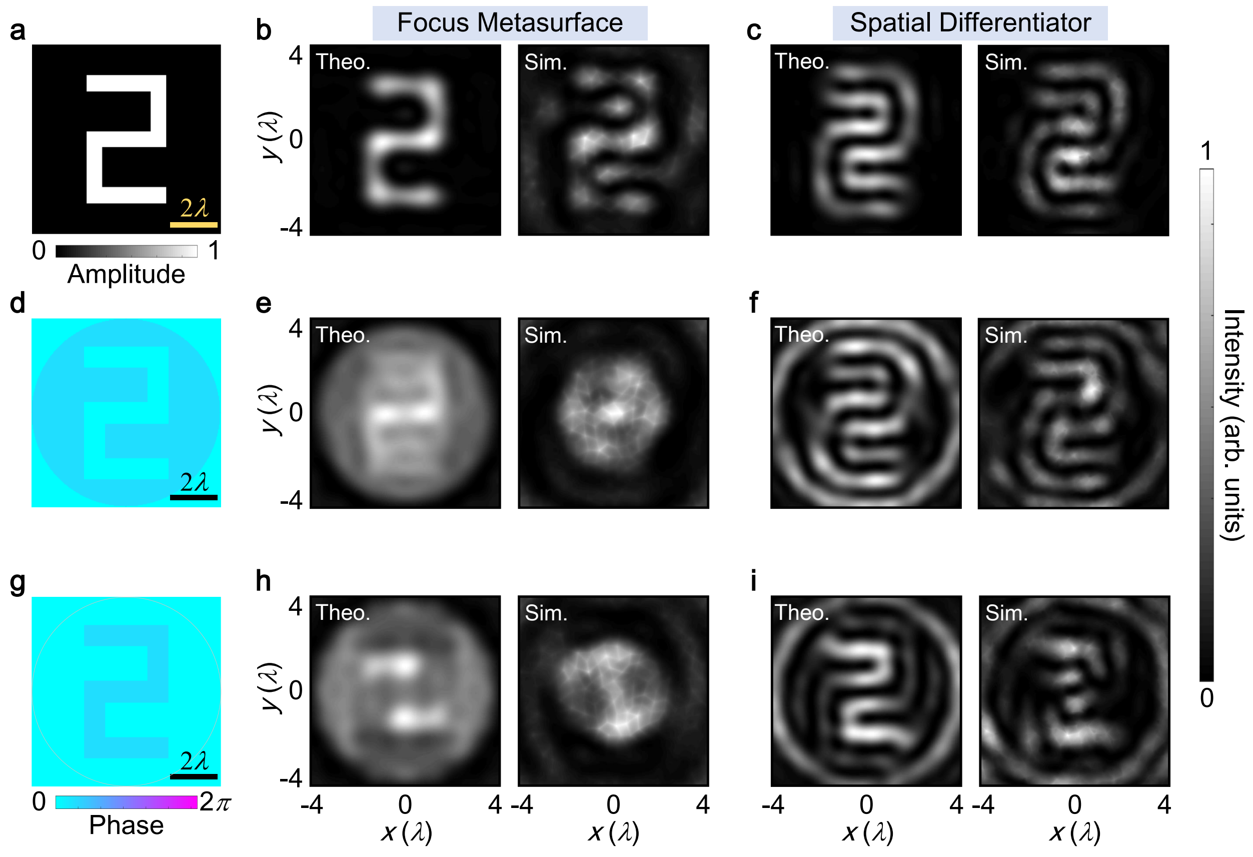

Supplementary Figure 21. **Comparison between focus metasurface and 2D spatial differentiator. a** An amplitude object in the shape of numeral 2 with a height of 6λ, a width of 3.75λ, and a slit of 0.75λ. Calculated and simulated intensity profiles of the amplitude object at the output plane as processed by (**b**) focus metasurface and (**c**) 2D spatial differentiator. **d** A phase object of numeral 2 of the same size illuminated by the Gaussian plane wave in 8λ width with a phase advance of $\pi/4$. Calculated and simulated intensity profiles of the phase object at the output plane based on (**e**) focus metasurface and (**f**) 2D spatial differentiator. **g-i** Same as (**d**)-(**f**), but for a phase object of numeral 2 with a phase advance of $\pi/4$ relative to the incident Gaussian plane wave.

Supplementary Note 23. Edge-enhanced imaging of 3D mini hands

Supplementary Figure **22**a shows photographs of the front and back views of a 3D mini hand with the forefinger stretched out. The scale bar represents a length of 3 mm. The incident acoustic field is a Gaussian plane wave launched by an ultrasonic transducer with a diameter of 12.7 mm operating at 1 MHz. The mini hand is made of PVC plastics, which provides phase contrast to the ambient medium. With the mini hand placed at the input plane, the measured acoustic intensity profile at the output plane is shown in Supplementary Fig. **22**b. It is observed that the outer edges of the 3D mini hand are enhanced, and the edge information of the forefinger is fully extracted. Additionally, the parts inside with enhanced edges are attributed to thickness differences within the mini hand. The intensity profile along the horizontal direction of the measured result is presented in Supplementary Fig. **22**c, revealing detailed edge information of the forefinger with a finger width of 2.0 mm.

Furthermore, we discuss a 3D mini hand with both the forefinger and midfinger stretched out, as depicted in the photographs of the front and back views in Supplementary Fig. **22**d. Supplementary Figure **22**e displays the measured acoustic intensity profile at the output plane. The boundaries of the mini hand are outlined, showcasing the edge-enhanced imaging of both the forefinger and midfinger. Meanwhile, the intensity profile along the horizontal direction of the measured result is illustrated in Supplementary Fig. **22**f, where the finger widths of the forefinger and midfinger are 1.5 mm and 1.9 mm, respectively. In this case, the 2D spatial differentiator is capable of detecting 3D phase objects with edge information extraction, which holds significance for practical applications in medical diagnosis and biological detection.

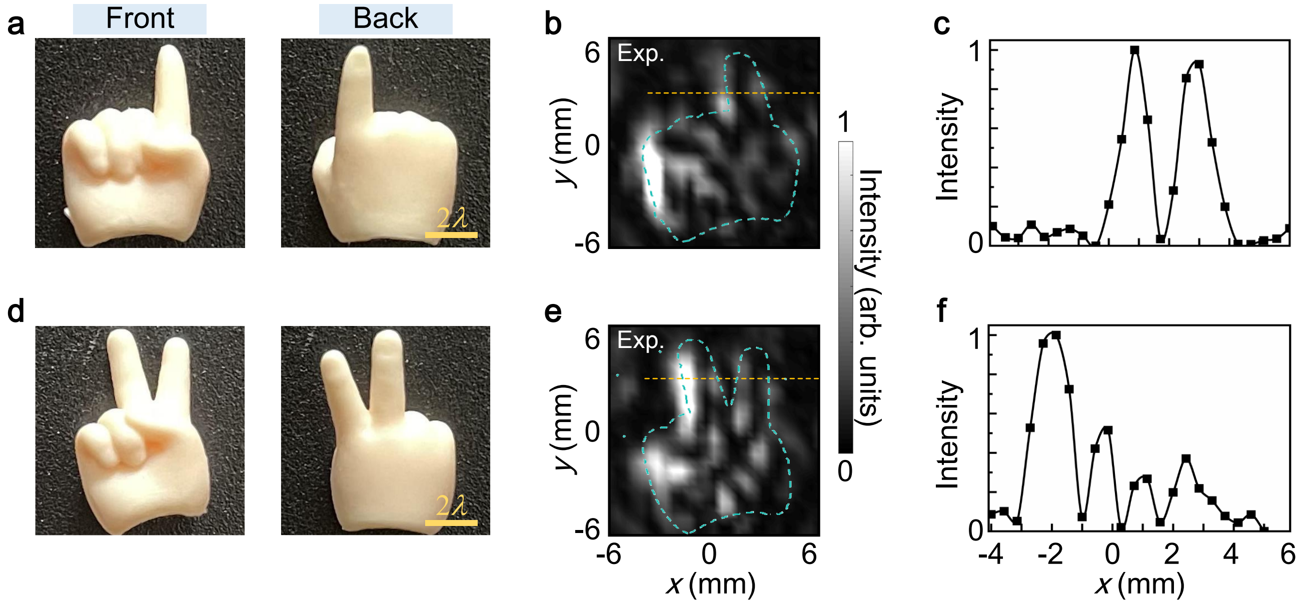

Supplementary Figure 22. **Edge-enhanced imaging of 3D mini hands. a** Photographs of the front and back views of a 3D mini hand with the forefinger stretched out. The scale bar represents a length of 3 mm. **b** Measured intensity profile at the output plane. **c** Intensity profile along the horizontal direction of experimental result. **d**-**f** Same as (**a**)-(**c**), but for a 3D mini hand with both the forefinger and midfinger stretched out.

Supplementary Note 24. 3D object imaging based on acoustic meta-differentiator

In the imaging system, we consider a 3D object composed of Slice I and Slice II, aligned with a separation distance of d along the z direction, as visually represented in Supplementary Fig. **23**a. Slice I takes on the form of numeral 2, possessing dimensions of 6λ in height, 3.75λ in width, and a line width measuring 0.75λ. On the other hand, Slice II shows the distinct shape of numeral 5, sharing identical dimensions as Slice I. When the distance f_1 is precisely 40λ, and the separation between these two slices is $d = 3\lambda$, that is, Slice I resides at the focal point of imaging system. In Supplementary Fig. **23**b, we present simulated acoustic intensity profiles at the imaging plane at $f_2 = 40\lambda$. It is evident that the images of numerals 2 and 5 significantly overlap, posing challenges for the observation of 3D structures. Furthermore, when we modify the distance f_1 to 37λ and position Slice II at the focal point, the corresponding simulated results shown in Supplementary Fig. **23**c also display interference from both slice structures, rendering it challenging to distinguish between Slice I and Slice II. Hence, our meta-differentiator might face challenges when employed for structural slice imaging of 3D objects, exhibiting significant overlapping features within the focal length.

Additionally, we discuss the scenario wherein two slices are separated by a large axial distance. For illustration, we modulate f_1 to 40λ, and Slice I and Slice II are aligned and positioned at 40λ and 45λ, respectively, with an axial separation of 5λ. In Supplementary Fig. **23**d, we present the corresponding simulated intensity profiles at the imaging plane of $f_2 = 40\lambda$. Notably, the numeral 2 is easily discerned and faithfully reconstructed, with all edges prominently enhanced at the imaging plane. When we displace the meta-differentiator away from Slice I by a distance of $f_1 = 35\lambda$, then Slice II effectively occupies the focal position. Supplementary Figure **23**e illustrates the corresponding simulated results at the imaging plane. In this scenario, all edges of the numeral 5 are accurately extracted and distinguishable from the numeral 2. The boundaries of both slice structures are distinctly delineated, underscoring the robust performance of our meta-differentiator in 3D object imaging across different planes. Consequently, our meta-differentiator proves suitable for the edge detection of 3D objects with large separation between the interior slice structures along the focal length. Furthermore, through optimizing the acoustic meta-differentiator, it is possible to reduce the focal length of imaging system, holding promise for the detection of adjacent slices within 3D objects. In summary, our method is well-suited for the edge detection of 3D objects featuring sparse interior slice structures, but may have limitations when applied to 3D object imaging characterized by densely packed slice structures. Ongoing investigations are dedicated to addressing the edge detection of more complex 3D objects.

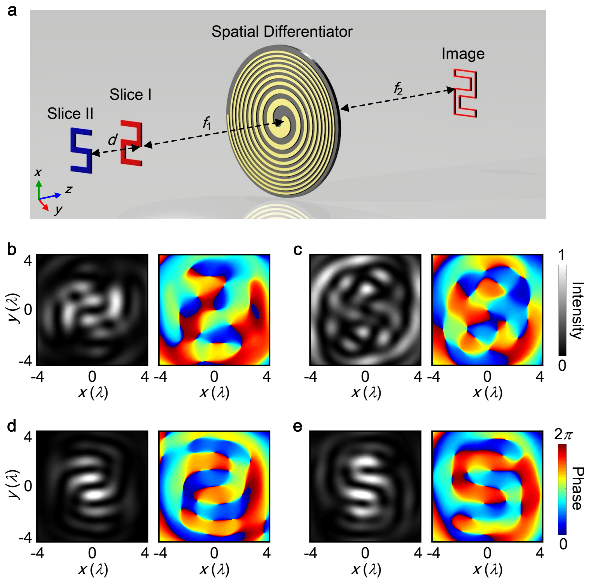

Supplementary Figure 23. **3D object imaging based on acoustic meta-differentiator.** **a** Schematic of 3D object imaging process in the imaging system employing an acoustic meta-differentiator. The 3D object consists of Slice I and Slice II. **b** Simulated acoustic intensity and phase distributions at the imaging plane $f_2 = 40\lambda$ with Slice I and Slice II positioned at 40λ and 43λ away from the meta-differentiator, respectively. **c** Similar to (**b**), but Slice I and Slice II placed at 37λ and 40λ, respectively. **d** Analogous to (**b**), but Slice I and Slice II positioned at 40λ and 45λ, respectively. **e** Equivalent to (**c**), but Slice I and Slice II located at 35λ and 40λ, respectively.

Supplementary Note 25. Comparison among prior acoustic meta-differentiators

We have conducted quantitative comparisons with the prior researches to underscore the enhancements and advantages of our proposed acoustic meta-differentiator. We have compiled a comprehensive overview of key attributes in Supplementary Table I, summarizing the reported acoustic meta-differentiators found in the prior literature sources [11–15]. To facilitate comparison, we present information on the working principle, working mode, device component, working condition, working medium, target objects, imaging dimension, 2D image demonstration, resolution, experimental validation, and measurement method.

Supplementary Table I. Comparison among prior acoustic meta-differentiators

**Literature**	**Zuo et al.**[11]	**Zuo et al.**[12]	**Zangeneh-Nejad et al.**[13]	**Molerón et al.**[14]	**Ma et al.**[15]	**This work**
**Working principle**	Fourier spatial filtering	Fourier spatial filtering	Green's function	Trapped resonance	Fourier spatial filtering	Fourier spatial filtering
**Working mode**	Transmission	Reflective	Reflective	Transmission	Transmission	Transmission/ Reflective
**Device component**	Three layers	Two layers	Single layer	Single layer	Two layers	Single layer
**Working condition**	Waveguide	Waveguide	Waveguide	Waveguide	Waveguide	Free space
**Working medium**	Air	Air	Air	Air	Air	Water
**Wavelength (mm)**	171.5	85.75	900	44.3	38.1	1.5
**Target objects**	Amplitude	Amplitude	Amplitude	Amplitude	Amplitude	Amplitude/ Phase
**Imaging dimension**	1D	1D	1D	1D	1D	2D
**2D imaging demonstration**	No	No	Yes	Yes	No	Yes
**Resolution (mm)**	130	100	Not reported	10	9.5	1.125
**Experimental validation**	No	No	No	Yes	Yes	Yes
**Measurement method**	No	No	No	Point-by-point scanning	Spatial spectrum superposition	Direct imaging

The transverse analysis reveals that several pioneering spatial differentiation techniques involve the utilization of a single planar metasurface in the Fourier domain for spatial filtering, accompanied with one or two additional metasurfaces to execute Fourier transforms on the input and output signals. This configuration results in an expanded overall system, potentially limiting the feasibility of compact system integration. In contrast, our proposed method leverages a single planar metamaterial for conducting differentiation operations directly on input signals, leading to a significant reduction in overall system dimensions and enhancing compatibility with compact integrated systems. Meanwhile, our structure can be employed in both transmission- and reflective-mode imaging systems, further expanding the application scope of computational metamaterials. In addition, most metamaterials are confined to operation within waveguides, constraining their broader applicability. Instead, our structure operates in free space, rendering it more flexible and suitable for practical use.

In light of working medium, the predominant focus of existing research has primarily revolved around edge detection of amplitude objects in air, with limited exploration into the realm of edge detection within underwater environments. Given the burgeoning applications in the domain of medical diagnostics, where tissues and organs are frequently treated as phase objects in liquid medium, the detection of phase object edges remains an unexplored facet within the computational metamaterials literature. Addressing this significant gap, our work presents a methodology for the efficient extraction of edge information from both amplitude and phase objects in liquid. This advancement holds substantial promise for advanced medical imaging applications. Additionally, it is worth emphasizing that the majority of contemporary demonstrations in spatial differentiation are primarily limited to 1D scenarios, resulting in edge enhancements along either the x or y direction, thus yielding anisotropic edge enhancements that may not be ideal for imaging applications. Achieving 2D edge enhancements, which encompass both horizontal and vertical edges simultaneously, typically necessitates the superposition of 1D edge-enhanced images along both the x and y directions. In contrast, our method inherently functions as a 2D spatial differentiator, a novel feature heretofore unreported in acoustics, directly enabling the simultaneous extraction of all object boundaries.

Furthermore, considering the intricate design inherent in existing methodologies, limited efforts have been dedicated to experimental validation of computational metamaterial performance. The extraction of edges through trapped resonances often mandates a laborious point-by-point scanning approach along both horizontal and vertical axes, resulting in a time-intensive process that lacks the ability to deliver real-time object edge information. Meanwhile, employing multiple metastructures to individually capture high-frequency components of objects and subsequently reconstruct edge contours through spatial spectrum superposition proves to be a complex strategy that falls short in promptly revealing distinctive object characteristics. In contrast, our proposed method streamlines the processing of acoustic signals, enabling the direct reconstruction of edge-enhanced images at the imaging plane without the need for intricate post-processing procedures. This approach offers a timely imaging solution, seamlessly integrated into the observation of dynamic object motions, thus presenting a marked advancement in the imaging field.

References

[1] Fu, W. W. *et al.* Ultracompact meta-imagers for arbitrary all-optical convolution. *Light: Sci. Appl.* **11**, 62 (2022).
[2] Wang, Z. C. *et al.* Single-layer spatial analog meta-processor for imaging processing. *Nat. Commun.* **13**, 2188 (2022).
[3] Kim, Y., Lee, G. Y., Sung, J., Jang, J. & Lee, B. Spiral metalens for phase contrast imaging. *Adv. Funct. Mater.* **32**, 2106050 (2022).
[4] Zhu, T. F. *et al.* Topological optical differentiator. *Nat. Commun.* **12**, 680 (2021).
[5] Melde, K., Mark, A. G., Qiu, T. & Fischer, P. Holograms for acoustics. *Nature* **537**, 518 (2016).
[6] Ritsch-Marte, M. Orbital angular momentum light in microscopy. *Philos. Trans. R. Soc. A* **375**, 20150437 (2017).
[7] Jesacher, A., Furhapter, S., Bernet, S. & Ritsch-Marte, M. Shadow effects in spiral phase contrast microscopy. *Phys. Rev. Lett.* **94**, 233902 (2005).
[8] Yu, X. M., Trallero-Herrero, C. A. & Lei, S. T. Materials processing with superposed bessel beams. *Appl. Surf. Sci.* **360**, 833–839 (2016).
[9] Liu, E. *et al.* Design of acoustic fresnel zone plate for contact stress measurement of aero-engine rotor mating surface. *J. Sound Vib.* **500**, 116035 (2021).
[10] Yu, G. K., Zou, X. Y. & Wang, P. F. Achromatic acoustic generalized phase-reversal zone plates. *New J. Phys.* **24**, 083009 (2022).
[11] Zuo, S. Y., Wei, Q., Cheng, Y. & Liu, X. J. Mathematical operations for acoustic signals based on layered labyrinthine metasurfaces. *Appl. Phys. Lett.* **110**, 011904 (2017).
[12] Zuo, S. Y., Tian, Y., Wei, Q., Cheng, Y. & Liu, X. J. Acoustic analog computing based on a reflective metasurface with decoupled modulation of phase and amplitude. *J. Appl. Phys.* **123**, 091704 (2018).
[13] Zangeneh-Nejad, F. & Fleury, R. Performing mathematical operations using high-index acoustic metamaterials. *New J. Phys.* **20**, 073001 (2018).
[14] Moleron, M. & Daraio, C. Acoustic metamaterial for subwavelength edge detection. *Nat. Commun.* **6**, 8037 (2015).
[15] Ma, C., Kim, S. & Fang, N. X. Far-field acoustic subwavelength imaging and edge detection based on spatial filtering and wave vector conversion. *Nat. Commun.* **10**, 204 (2019).
